# Proton-shuttling nanosheet membranes enable high-power-density protonic fuel cells

**DOI:** 10.1126/sciadv.aea1569

**Published:** 2026-05-15

**Authors:** Kaiqiang He, Yuxiang Wang, Dehua Dong, Fanmengjing Wang, Kevin Ung, Zhuyuan Wang, Zhikao Li, Xiwang Zhang, Shanwen Tao, Jacek J. Jasieniak, Paul A. Webley, Douglas R. MacFarlane, Jefferson Zhe Liu, Zongping Shao, Huanting Wang

**Affiliations:** ^1^ Department of Chemical and Biological Engineering, Monash University, Clayton, VIC 3800, Australia.; ^2^ UQ Dow Centre for Sustainable Engineering Innovation, School of Chemical Engineering, The University of Queensland, St Lucia, QLD 4072, Australia.; ^3^ School of Engineering, University of Warwick, Coventry CV4 7AL, UK.; ^4^ Department of Materials Science and Engineering, Monash University, Clayton, VIC 3800, Australia.; ^5^ School of Chemistry, Monash University, Clayton, VIC 3800, Australia.; ^6^ Department of Mechanical Engineering, The University of Melbourne, Parkville, VIC 3010, Australia.; ^7^ WA School of Mines: Minerals, Energy and Chemical Engineering (WASM-MECE), Curtin University, Perth, WA 6102, Australia.

## Abstract

High-temperature operation enhances the efficiency and design simplicity of electrochemical devices, but conventional polymer membranes lose proton conductivity rapidly due to dehydration. Atomically thin nanosheets can selectively transport thermal protons through nanoscale corrugations and quantum tunneling, making them promising for high-temperature proton-conducting membranes. However, stacked nanosheet assemblies often suffer from poor proton transport between layers. We built nanosheet-based membranes by bridging individual nanosheets using nanoconfined phosphoric acid. This architecture enables low-tortuosity, synergistic proton transport via both through-nanosheet conduction and hydrogen bond–mediated hopping along confined acid layers, resulting in ultrafast, stable proton conduction under anhydrous high-temperature conditions. A polyethylenimine-functionalized graphene/boron nitride bilayer membrane achieves a proton conductivity of 166 millisiemens per centimeter and delivers a power density of 1011 milliwatts per square centimeter in hydrogen fuel cells at 250°C, outperforming most previously reported anhydrous proton-conducting membranes. Furthermore, it exhibits superior methanol tolerance, achieving 502 milliwatts per square centimeter on concentrated methanol. This work offers a versatile platform for next-generation high-temperature proton-conducting membranes.

## Teaser

Protonic nanosheet–assembled membranes with nanoconfined phosphoric acid unlock ultrafast proton transport at 250°C.

## INTRODUCTION

Proton-conducting membranes are essential components in electrochemical systems such as fuel cells (*1*), water electrolysis (*2*), CO_2_ reduction (*3*), and ammonia synthesis (*4*), enabling fast proton transport between electrodes while maintaining the separation of half-cells. Proton-exchange membranes (PEMs), such as perfluorosulfonic acid polymers, exhibit excellent proton conductivity when fully hydrated and have been widely used for low-temperature devices (*5*). However, current PEMs rely on a high degree of hydration to form proton-conducting networks, which limits their applicability at high temperatures due to dehydration. It is well-known that increasing the operating temperature of PEM fuel cells (PEMFCs) to above 100°C not only can enhance the device performance but also can simplify the system design (*6*). Inorganic acids, such as solid acids and phosphoric acid (PA), exhibit high proton conductivity at temperatures above 150°C (*7*–*9*). Solid acid fuel cells have demonstrated a power density of 415 mW cm^−2^ at 240°C (*10*). However, a steam supply is required to suppress the dehydration of the compounds at elevated temperatures (*11*). Alternatively, PA has been doped into polymers such as polybenzimidazole (PBI) to prepare proton-conducting membranes (*12*). PA-doped PBI membrane fuel cells can achieve a power density of 536 mW cm^−2^ at 160°C (*13*), but they suffer from rapid performance degradation due to extensive acid loss when operated above 180°C (*14*). These exciting developments and remaining challenges motivate us to explore alternative materials for constructing proton-conducting membranes for high-temperature applications.

The recent discovery of high proton conductivity in some two-dimensional (2D) monolayer nanosheets presents a promising class of protonic materials for high-temperature proton-conducting membranes (*15*–*17*). These atomically thin crystals uniquely allow thermal protons to pass through while blocking fuel molecules (fig. S1A), offering a rare combination of high conductivity and selectivity (*18*). Two key mechanisms have been proposed to explain the facilitated proton permeation through the electron cloud vacancies in hexagonal rings in these nanosheets. First, nanoscale corrugation, arising from the inherent flexibility of 2D materials and thermally induced distortions at elevated temperatures, can locally reduce the energy barrier for proton transport (*19*, *20*). Besides, the low mass of protons enables quantum nuclear effects, such as quantum tunneling, allowing protons to penetrate the nanosheet rings probabilistically without overcoming the energy barriers (*18*). Notably, proton transport directly through the monolayer nanosheet provides a highly efficient, low-tortuosity pathway. In contrast, membranes constructed from widely used multilayered nanosheets, which are impermeable to protons, primarily rely on in-plane proton conduction along surface functional groups, leading to increased path tortuosity and hence hindering through-membrane conduction (*21*, *22*). Current efforts to transfer large-area monolayer nanosheets onto polymer surfaces face challenges in nanosheet handling and their high-temperature applications are limited by the thermal stability of polymers (*23*–*25*). Although nanosheets can be readily stacked to form membranes with favorable mechanical properties, such assemblies show a marked increase in proton transport resistance between the nanosheets due to lattice mismatch and long transport distances (*26*, *27*). As a result, despite the exceptional potential of through-nanosheet proton transport, realizing this mechanism in macroscale membranes suitable for practical fuel cell applications remains a major challenge.

**Fig. 1.**
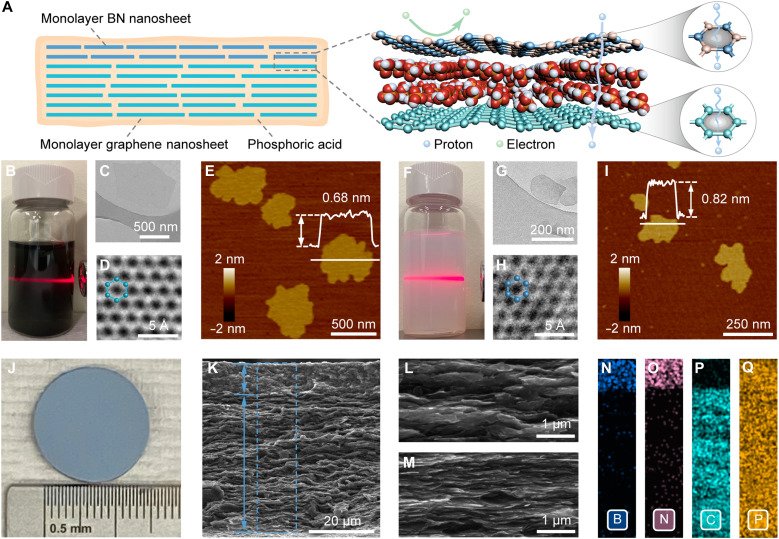
The materials and structures of the 2D protonic nanosheets based membrane. (**A**) Schematic illustration showing a bilayer structure of the graphene/BN/PA (GBP) membrane for proton transport. Proton-conducting BN layer is used as an electron insulator to minimize the electrical conductivity of the membrane. At elevated temperatures, protons can permeate through the expanded electron cloud vacancies within the hexagonal rings of monolayer graphene and boron nitride nanosheets. (**B**) Photograph of monolayer PEI-graphene nanosheets dispersed in water showing the Tyndall effect. (**C**) Transmission electron microscopy (TEM) and (**D**) high-resolution transmission electron microscopy (HR-TEM) images of monolayer PEI-graphene nanosheets. (**E**) Atomic force microscopy (AFM) image of monolayer PEI-graphene nanosheets with height profile inserted. (**F**) Photograph of monolayer PEI-BN nanosheets dispersed in water showing the Tyndall effect. (**G**) TEM and (**H**) HR-TEM image of monolayer PEI-BN nanosheets. (**I**) AFM image of monolayer PEI-BN nanosheets with height profile inserted. (**J**) Photograph of a free-standing GBP membrane. (**K**) Scanning electron microscopy (SEM) cross-sectional image of a typical GBP membrane. Locally magnified SEM cross-sectional images of the top BN layer (**L**) and bottom graphene layer (**M**), respectively, within the GBP membrane. (**N** to **Q**) EDX maps showing the distribution of boron (N), nitrogen (O), carbon (P), and phosphor (Q) within the blue square in (K), respectively.

Herein, we report a strategy to fabricate highly proton-conductive single-layer nanosheets into membranes with high anhydrous proton conductivity by bridging the nanosheets using inorganic proton conductors. Monolayer polyethylenimine-functionalized graphene (PEI-graphene) and hexagonal boron nitride (PEI-BN) nanosheets are used as the building blocks, with PA as an example of inorganic proton conductors to illustrate the concept (Fig. 1A). The membrane design encompasses four main considerations: (i) using 2D protonic nanosheets as proton-permeable building blocks to facilitate through-plane proton transport; (ii) assembling 2D nanosheets to form 2D nanochannels, which are crucial for nanoconfinement and stabilization of PA at elevated temperature; (iii) nanoconfined PA occupies intersheet spacings, enabling intersheet proton transport while blocking permeation of fuel molecules (fig. S1B); and (iv) combining the electrically insulating nature of boron nitride (BN) nanosheets and excellent mechanical properties of graphene nanosheets (*28*), which leads to a bilayer structured membrane with a thin BN layer to block electron transfer and a graphene layer to provide sufficient mechanical strength. To demonstrate the efficacy of the design, we apply it to PEMFCs, where the membrane based on 2D protonic nanosheets offers ultrafast nanosheet-PA synergistic proton transport and high acid retention through nanoconfinement.

## RESULTS

### Membrane fabrication and characterizations

Monolayer PEI-graphene and PEI-BN nanosheets were prepared via sticky ball milling using polyethylenimine (PEI) to assist exfoliation (Fig. 1, B and F, and fig. S2) (*29*). High-resolution transmission electron microscopy (HR-TEM) image reveals the honeycomb lattice of graphene with a 1.44-Å C─C distance (Fig. 1D), matching the {2110} facets in the selected-area electron diffraction (SAED) pattern (fig. S3, A and B). Monolayer graphene is confirmed by the stronger {1100} diffraction spots relative to the {2110} spots in the SAED pattern (*30*). Based on atomic force microscopy (AFM) statistical analysis, the PEI-graphene nanosheets have an average lateral size of 0.76 μm, an average thickness of 0.68 nm, and a monolayer ratio of ~95% (Fig. 1E and fig. S4, A and B). Similarly, the PEI-BN nanosheets show a hexagonal lattice with a 1.45-Å B─N spacing (Fig. 1H), an average lateral size of 0.25 μm, an average thickness of 0.82 nm, and a monolayer ratio of ~85% (Fig. 1I and figs. S3, C and D, and S4, C and D). Fourier transform infrared (FTIR) spectra present N─H bending from PEI, indicating its attachment to the nanosheet surfaces (fig. S5A). Thermogravimetric analysis (TGA) reveals PEI decomposition begins around 300°C, with grafting amounts on PEI-graphene and PEI-BN nanosheets estimated at 7.2 and 5.5 wt %, respectively (fig. S5B). Moreover, both nanosheets exhibit good thermal stability in air. In particular, PEI-graphene remains stable within the operating temperature range of this work (≤250°C), as further confirmed by the isothermal TGA measurements (fig. S5C), while PEI-BN shows stability at even higher temperatures.

**Fig. 2.**
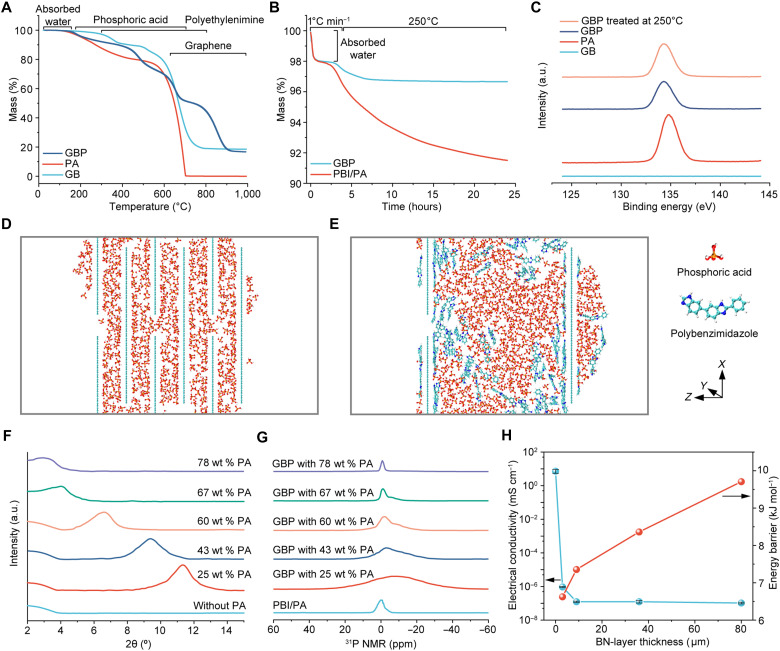
Physiochemical property characterization of the GBP membrane. (**A**) TGA curves of PA, the graphene/BN (GB) membrane, and GBP membrane in air. (**B**) Recorded mass loss of GBP membrane when heated at a rate of 1°C min^−1^ to 250°C and kept at 250°C in air for 24 hours. A pretreatment at 100°C for 3 hours was conducted to minimize moisture influence on the TGA tests. The mass loss observed below 200°C is primarily due to the evaporation of absorbed water from the air. (**C**) XPS P 2p spectra of GB membrane, PA, GBP membrane, and GBP membrane after treatment at 250°C in air for 24 hours. MD simulation snapshots showing the PA loss in the slit structure of the PA-doped nanosheet membrane (**D**) and the box structure of the PA-doped PBI membrane (**E**) after a 10-ns NVT run at 250°C. (**F**) XRD patterns of GB membranes with different amounts of PA incorporated. (**G**) Solid-state ^31^P nuclear magnetic resonance (NMR) spectra of PA-doped PBI membrane and GBP membranes with varied PA loading. (**H**) Electrical conductivity and energy barrier for proton transport in GBP membrane with different BN layer thicknesses. a.u., arbitrary units.

The 2D protonic nanosheet membranes were prepared using vacuum filtration, sequentially depositing PEI-graphene and PEI-BN nanosheet solutions onto a porous polymer substrate, followed by vacuum drying and PA incorporation (fig. S6). The produced graphene/BN/PA (GBP) membrane has a typical bilayer structure, which comprises a 9-μm top PEI-BN layer and a 41-μm bottom PEI-graphene layer (Fig. 1, J and K). The bottom PEI-graphene layer exhibits a better-aligned laminal structure and higher packing density due to its larger aspect ratio (~1120) compared to the PEI-BN nanosheets (~300) (Fig. 1, L and M). The ordered stacking of the nanosheets benefits the formation of a dense membrane for fuel cell applications requiring a low fuel crossover and high mechanical stability. Energy-dispersive x-ray spectroscopy (EDX) confirms the bilayer structure, with boron and nitrogen in the top PEI-BN layer and carbon in the bottom PEI-graphene layer (Fig. 1, N to P). The uniform distribution of phosphor indicates that the PA has been evenly incorporated into the membrane (Fig. 1Q). The PA content is estimated at 59 wt % from EDX (fig. S7), consistent with 61 wt % from TGA results (Fig. 2A).

The thermal degradation of PA starts at about 165°C in the air, losing water and forming metaphosphoric acid (*31*). In the GBP membrane, strong interactions between PA and PEI molecules in 2D nanochannels enhance the thermal stability of PA, PEI, and graphene, with decomposition onset temperatures of 225°, 437°, and 802°C, respectively. As a result, the GBP membrane shows excellent thermal stability, with only 2.1% PA loss at 250°C compared to 10.3% in a conventional PBI/PA membrane (Fig. 2B). FTIR and nuclear magnetic resonance (NMR) analyses confirm no noticeable structural degradation at 250°C (fig. S8). X-ray photoelectron spectroscopy (XPS) analysis shows a shift in the P 2p peak due to PA’s interaction with PEI (Fig. 2C), and minimal changes in peak ratios after heat treatment further validate the thermal stability of the GBP membrane (table S1). Additionally, thermal treatment to remove 85% of PEI from the PEI-functionalized graphene/BN (PEI-GB) membrane results in greater PA loss (fig. S10, B and C), indicating that PEI enhances PA retention through chemical interactions.

To reveal the contribution of nanoconfinement to the enhanced thermal stability of the GBP membrane, molecular dynamics (MD) simulations were performed to compare the confining effects of the nanosheet membrane’s slit-like structure and the PBI membrane’s box structure (Fig. 2, D and E, and fig. S12). At room temperature, both structures demonstrate good retention of PA and water with minimal mass loss (fig. S13A). However, when the molecular motion is intensified at 250°C, the nanosheet membrane exhibits superior PA retention due to strong capillary forces within the 2D nanochannels as compared to the amorphous PBI with randomly arrayed molecular chains having less steric hindrance to the PA movement (fig. S13B). Notably, the nanosheets within the slit-like structure can break down the bulk PA into smaller segments, constraining the motion of PA molecules perpendicular to the nanosheets. This further confirms the membrane’s potential for effectively maintaining PA at elevated temperatures.

The intersheet spacings of the GBP membranes increase with higher PA loading, indicated by x-ray diffraction (XRD) peak shifts to lower angles (Fig. 2F, fig. S14, and table S2). This expansion increases membrane thickness and slightly reduces density (fig. S15J), as PA fills interlayer gaps. The PA-filled GBP membrane also demonstrates extremely low gas permeability (fig. S14G), confirming its dense structure. However, greater spacing slightly increases PA loss at 250°C due to reduced nanoconfinement effect (fig. S16). This aligns with the MD simulations, highlighting the critical role of nanoconfinement in PA retention within the GBP membrane. In the solid ^31^P NMR spectra (Fig. 2G), higher PA loading increases the normalized peak area of weakly absorbed PA owing to expanded channels and excess PA (fig. S18A). Furthermore, the corresponding chemical shifts also rise, reflecting reduced shielding in the expanded channels (fig. S18B). Besides of PA loading, the BN-layer thickness also influences membrane properties. The role of the BN layer is to block electron conduction through the membrane and provide nanoconfined proton transport. With a 9-μm-thick BN layer, the cross-membrane electron transport was effectively blocked to have an extremely low electrical conductivity of around 1.25 × 10^−7^ mS cm^−1^ (Fig. 2H).

To highlight the advantages of the bilayer design, membranes with uniformly mixed PEI-BN and PEI-graphene nanosheets were also prepared (fig. S19, A to C). Due to similar surface functionality with PEI, BN, and graphene nanosheets mixed uniformly at various ratios, forming homogeneous structures confirmed by the even distribution of carbon and boron elements in EDX maps (fig. S19, D to G). Membranes with BN-to-graphene ratios of 1:4 and 1:1 showed good bending flexibility, while the 4:1 ratio membrane cracked with slight bending, indicating weaker mechanical strength. As BN content increased, electrical conductivity decreased (fig. S19H) but remained higher than the 9.4 × 10^−7^ mS/cm in the bilayered membrane with a 3-μm BN layer. This demonstrates that the bilayer GBP design is more effective in achieving high electrical insulation and robust mechanical properties for our nanosheet membranes.

### Proton transport properties

**Fig. 3.**
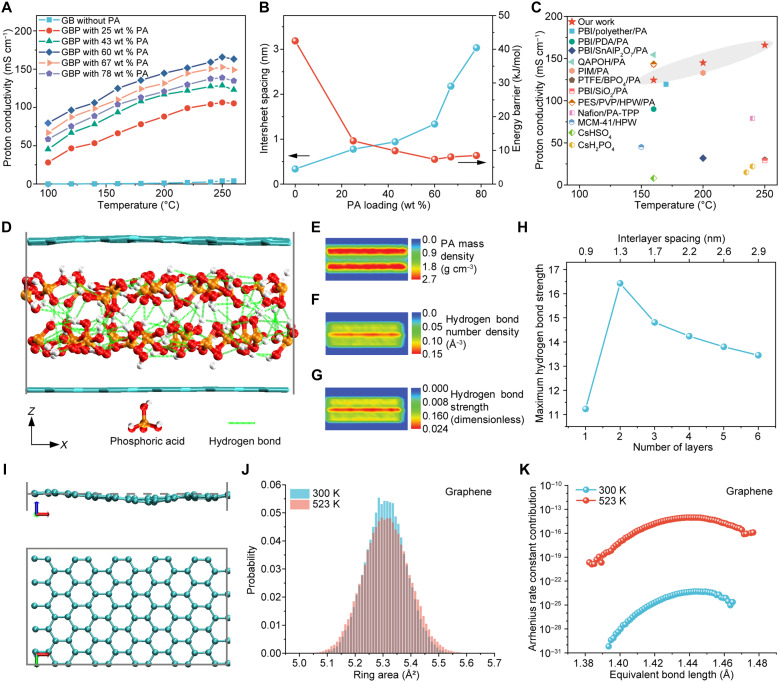
Proton transport properties and mechanisms of the GBP membranes. (**A**) Temperature-dependent through-plane proton conductivity of GB membrane, GBP membranes with 25, 43, 60, 67, and 78 wt % PA incorporated. (**B**) Intersheet spacing of nanosheets and energy barrier for proton transport of GBP membranes with varied PA loading. (**C**) Proton conductivity comparison of the 60 wt % PA-doped and 50-μm-thick GBP membrane with the state-of-the-art membranes at various temperatures (also see table S4). (**D**) Schematic illustration of MD simulation for hydrogen bond network when two PA layers are formed between the nanosheets at 250°C. (**E** to **G**) Distribution maps of PA mass density (E), hydrogen bond number density (F), and hydrogen bond strength (G) corresponding to (D); see the “Hydrogen bond calculations” section for definitions. (**H**) Variation of the maximum hydrogen bond strength with the number of PA layers formed between the nanosheets from the MD simulation. (**I**) Schematic illustration of the fluctuation of graphene nanosheet at 523 K with reference line showing the initial state. (**J**) Comparison of the ring area distribution of graphene nanosheet at 300 and 523 K. The relevant data for BN are provided in fig. S38 (C and D). (**K**) Comparison of Arrhenius rate constant contribution of different equivalent bond length for graphene at 300 and 523 K.

The through-plane proton conductivities of the GBP membrane were measured along its thickness direction from 100° to 260°C with varying PA loadings and nanosheet layer thicknesses (Fig. 3A; fig. S21, A and B; and table S3). Compared to the PA-free GB membrane, the GBP membranes present notably higher proton conductivity and lower energy barriers due to reduced transport resistance within the PA-filled intersheet spacing (Fig. 3B). The proton conductivity increases with PA loading up to 60 wt %, reaching a peak of ~166 mS cm^−1^ at 250°C. A slight decline at 67 wt % is likely attributed to the enlarged nanochannel size, indicating 60 wt % PA loading as optimal for fast intersheet proton conduction.

The proton conductivities of the GBP membranes decrease with increasing BN layer thickness (fig. S21A), aligning with a rising energy barrier. The GBP membrane with a 3-μm-thick BN layer showed the highest proton conductivity, comparable to the pure 41-μm-thick graphene/PA membrane (~172 mS cm^−1^, fig. S23A). However, its relatively high electrical conductivity and poor mechanical strength risked short-circuiting during fuel cell tests. Considering its electrical resistivity and structural stability, the 9-μm-thick BN layer was selected as optimal for the GBP membrane. Similarly, increasing graphene layer thickness reduces proton conductivity (fig. S21B). The GBP membrane with a 15-μm-thick graphene layer exhibited the highest proton conductivity, which is close to that of the pure 9-μm-thick BN/PA membrane (~186 mS cm^−1^, fig. S23B). However, it showed poor mechanical properties and was prone to cracking during fuel cell testing. The 41-μm-thick graphene layer provided robust mechanical strength and comparable proton conductivity, making it the optimal choice for fuel cell applications.

The effect of nanosheet geometry on the proton conductivity of GBP membranes was investigated by tuning the lateral size and thickness of PEI-graphene nanosheets through milling parameters, as described in our previous work (*29*). Increasing the milling time from 10 to 15 hours reduced the average lateral size of the PEI-graphene nanosheets from 760 to 210 nm while maintaining a high monolayer ratio (~97%) (fig. S24, A to C). The GBP membrane made from smaller monolayer PEI-graphene nanosheets showed minimal XRD peak shifts, indicating unchanged intersheet spacing and similar intersheet proton transport (fig. S24E). Only a slight (~6%) increase in proton conductivity was observed (fig. S24F), likely due to reduced pathway tortuosity caused by more gaps between nanosheets (fig. S24D) (*32*). However, the marginal increase in proton conductivity does not scale with the substantial reduction in tortuosity, which results from a 2.6-fold decrease in nanosheet size variation. This observation suggests that through-nanosheet proton transport plays an important role in proton conduction across the membrane.

The impact of nanosheet thickness on proton transport was examined by replacing monolayer PEI-graphene nanosheets with multilayered PEI-graphene nanosheets (~2 to 3 layers, ~840-nm lateral size), prepared via reduced exfoliation efficiency (fig. S25, A to C). Due to the staggered arrangement of neighboring nanosheets (fig. S25D), the multilayered graphene nanosheets are impermeable to thermal protons (*15*, *26*). Despite similar intersheet spacing (fig. S25E), the resulting GBP membrane showed ~24% lower proton conductivity (fig. S25F), likely due to blocked through-nanosheet proton transport. This suggests again that the through-nanosheet proton transport could help facilitate proton transport along the membrane’s thickness direction.

In addition, other proton transport properties including anisotropy and long-term stability were investigated. The through- and in-plane proton conductivities of the GBP membrane were measured along its thickness and plane directions, respectively. The higher in-plane proton conductivity is attributed to lower pathway tortuosity (fig. S26, A and B). The faster proton conduction along the plane direction reveals the proton prefers to transport along the 2D nanochannels, supported by a lower energy barrier (fig. S26C). Compared with other advanced membranes, the 60 wt % PA-doped, 50-μm-thick GBP membrane exhibits outstanding proton conductivity from 100° to 250°C, achieving among the highest reported values at 250°C (Fig. 3C). It also shows excellent thermal stability, with only a ~6 mS cm^−1^ drop after 120 hours at 250°C (fig. S27).

### Proton transport mechanisms

In the GBP membranes, PA molecules fill the intersheet spacing of protonic nanosheets, forming continuous proton transport pathways (fig. S28). There are two main proton transport mechanisms, leading to rapid and synergistic proton conduction. The first mechanism involves through-nanosheet proton transport, particularly at elevated temperatures (*15*, *19*). The other is the along-nanochannel proton hopping via the hydrogen bond network formed by nanoconfined PA molecules through the Grotthuss mechanism (*33*). Based on through-/in-plane proton conductivity measurements, the difference in their proton conductivities is much smaller than the difference in path tortuosity (fig. S26). Together with the nanosheet size and thickness effects, the through-nanosheet proton transport should play a critical role in facilitating proton transport through the membrane.

To investigate the nanoconfinement effect on along-nanochannel proton transport, full-atom MD simulations were conducted (figs. S29 to S32). As nanosheet spacing increased with varying PA loadings, confined PA molecules formed ordered lamellar structures with distinct hydrogen bond networks (figs. S31 and S32). The two-layer PA structure presented the highest hydrogen bond density and strength, favoring ultrafast proton transport along the nanosheets (Fig. 3, D to G, and figs. S31 to S34). This optimal structure, with an intersheet spacing of ~1.29 nm, correlated with the experimental results, where the GBP membrane with 60 wt % PA and 1.34-nm spacing exhibited the highest proton conductivity (Fig. 3, A and H). However, excess PA led to increased spacing and loss of the lamellar structure, resembling the bulk state of PA (figs. S31 and S32). Ab initio molecular dynamics (AIMD) simulations revealed that the two-layer PA structure achieved the longest free proton lifetime and highest free proton proportion (fig. S35C), supporting its highest proton conductivity. Given its strong alignment with experimental observations, the proton conductivity of the GBP membrane is strongly influenced by the hydrogen bond network formed by PA molecules. This suggests that the proton hopping along the hydrogen bond network in the nanochannels could also be an important mechanism for proton conduction within the membrane.

To gain a deeper understanding of the through-nanosheet proton transport at elevated temperatures, we performed additional characterizations and MD simulations. At 250°C, buckling ridges formed on monolayer graphene and BN nanosheets (fig. S36). These thermal distortions, similar to nanoscale wrinkles and strain, have been shown to markedly enhance proton transport through 2D crystals, as mapped by high-resolution scanning electrochemical cell microscopy (*19*). AIMD simulations suggested that the expanded rings on nanosheets reduce the energy barrier for through-nanosheet proton transport (Fig. 3I and figs. S37 to S40). As the temperature rises from 300 to 525 K, the ring area distribution shifts toward larger rings (Fig. 3J). The weighted proton transport rate constant through each ring area increases substantially from 300 to 523 K (Fig. 3K). The estimated increments in the overall proton transport probability with this mechanism are 10^9^-fold for graphene and 10^6^-fold for BN, respectively. It suggests that the proton transport through the corrugations of protonic nanosheets is substantially improved at elevated temperatures. This trend is consistent with the improved proton conductivity measured in the experiments from room temperature to 523 K, despite some overestimation (fig. S41A). Additionally, a recent theoretical study has proven quantum tunneling possibility is temperature-dependent and can be transformed into the form of the Arrhenius equation with a temperature-dependent preexponential factor and energy barrier (*34*). In fig. S41B, the Arrhenius rate constant for quantum tunneling exponentially increases from 300 to 523 K, achieving a 10^6^-fold increment. This trend aligns with the experimental measurements of proton conductivity, but a clear overestimation is also observed. Although both ring expansion simulations and quantum theory calculations show trends consistent with experimental results, they overestimate the temperature dependence of proton conductivity in our membranes. This discrepancy likely arises from the defects in the nanosheets produced via ball milling, as demonstrated in our previous work (*29*), whereas the models assume defect-free nanosheets. The comparative analysis of through-plane and in-plane proton transport, along with the impact of nanosheet thickness on proton transport, indicates that through-nanosheet and along-nanochannel transport mechanisms act synergistically. This synergy enhances overall proton conductivity by substantially reducing the tortuosity of proton transport pathways (fig. S28).

### Fuel cell performance

**Fig. 4.**
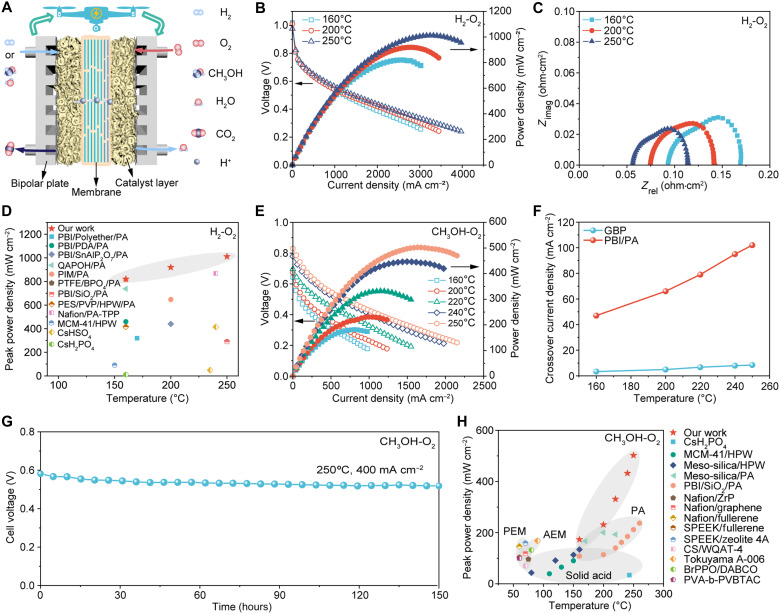
Fuel cell performance and durability of the GBP membrane. (**A**) Schematic illustrating a GBP membrane–based fuel cell powered by hydrogen or methanol, and proton conduction through the membrane. (**B**) Current-voltage (*I*-*V*) polarization and power density plots, and (**C**) electrochemical impedance spectra of the H_2_-O_2_ fuel cell assembled with 60 wt % PA-doped and 50-μm-thick GBP membrane measured at different temperatures. (**D**) Comparison of the maximum power density of the cell based on the GBP membrane with other advanced membrane-based cells under H_2_-O_2_ conditions (also see table S4). (**E**) *I*-*V* polarization and power density plots of the DMFC assembled with the GBP membrane measured at different temperatures and fed with oxygen at the cathode. (**F**) Methanol crossover current density of GBP- and PBI/PA-based MEAs measured at different temperatures. (**G**) Operating stability of the GBP membrane–based DMFC supplied with 16 M methanol and oxygen at a current density of 400 mA cm^−2^ and 250°C. (**H**) Comparison of peak power density of DMFC using the GBP membrane and other advanced membranes, including PEMs, anion-exchange membranes (AEM), solid acid–based membranes, and PA-based membranes (also see table S5).

The optimized GBP membrane (60 wt % PA, 50-μm thickness) was incorporated into a membrane electrode assembly (MEA) to evaluate its hydrogen fuel cell performance with Pt/C as electrocatalysts (Fig. 4A). The maximum power density increases from 160° to 250°C due to enhanced proton conductivity and improved electrocatalytic activity (Fig. 4, B and C). At 250°C, the membrane achieved an outstanding peak power density of ~1011 mW cm^−2^, three times higher than the PBI/PA membrane, owing to its higher proton conductivity (fig. S42, A and B). The GBP membrane also exhibited excellent long-term stability at 250°C, maintaining stable operation at 400 mA cm^−2^ with a low voltage decay rate of 0.19 mV hour^−1^ over 150 hours (fig. S43A). Posttest polarization and impedance measurements show only minor increases in ohmic and polarization resistance, and the cell retains 93% of its initial peak power density (fig. S43, B and C), confirming the robustness of the membrane under sustained high-temperature operations. This stability is crucial for the practical implementation of fuel cells and other electrochemical devices, highlighting the GBP membrane’s potential for reliable and long-term operation in high-temperature applications.

To evaluate the impact of the asymmetric membrane structure, fuel cell performance was tested with H_2_ flowing on both the graphene and BN sides (fig. S44). The minimal difference in peak power density suggests the asymmetry has little effect on the performance. Despite the different electronic conductivity and proton affinity of graphene and BN, both create similar 2D nanochannels that confine PA, promoting efficient proton transport and gas blocking. Redox reactions primarily occur at the three-phase interfaces involving catalysts, PA, and reacting gases. The asymmetric GPB membrane provides sufficient surface area for these reactions, ensuring comparable performance when switching the reacting gases. Compared with conventional polymer and ceramic protonic cells, the GBP membrane–based fuel cell demonstrates markedly higher power density at elevated temperatures (Fig. 4D). This superior performance is attributed to efficient proton conduction and high-temperature stability enabled by nanoconfinement.

The potential application of GBP membranes in direct methanol fuel cells (DMFCs) was also explored. DMFCs, using methanol from biomass or renewables, offer advantages in transport and storage over hydrogen. DMFCs particularly show great potential in portable devices due to methanol’s high energy density. However, due to the methanol crossover issue of Nafion and other polymer membranes, only highly diluted methanol (typically 1 to 2 M) can be used as fuel in conventional DMFCs (*35*, *36*), resulting in poor practical energy density. In addition, these DMFCs suffer from low power density and CO poisoning issues in the electrodes. Because monolayer BN and graphene nanosheets are only permeable to protons, methanol crossover can be minimized in GBP membranes prepared with closely stacked nanosheets. The GBP membrane–based DMFC exhibits an impressive peak power density of 502 mW cm^−2^ at 250°C when supplied with a high concentration of methanol (16 M) as fuel on the anode and oxygen on the cathode (Fig. 4E). With the rising temperature, the increase in peak power density can be attributed to the concurrent reduction in both ohmic and polarization resistance, coupled with an increase in open circuit potential (fig. S46). A temperature transition point occurs at ~200°C, where the rate of increase in peak power density changes from 1.45 to 5.33 mW cm^−2^ °C^−1^. This improvement can be ascribed to greatly enhanced kinetics for methanol oxidation at the anode, as evidenced by a substantial increase in open circuit potential and a reduction in polarization resistance (*37*).

Methanol crossover is indicated by the limiting current density resulting from methanol permeating from the anode to the cathode under an applied voltage. Owing to the well-aligned 2D nanochannels and dimensional stability, the GBP membrane displays much lower methanol crossover current density (3.1 to 8.4 mA cm^−2^) compared to the PBI/PA membrane (47 to 102 mA cm^−2^) between 160° and 250°C (Fig. 4F and fig. S47). In addition to its high proton conductivity, the GBP membrane’s resistance to methanol crossover preserves a high open circuit potential, resulting in superior DMFC performance compared to the PBI/PA membrane at elevated temperatures (fig. S48). Due to the strong methanol impermeability of the GBP membrane, raising the methanol concentration from 5 to 20 M has minimal impact on its open circuit voltage (fig. S49). The optimal performance is achieved at 16 M (with a methanol molar ratio of 0.45), which can be attributed to the 1:1 reaction of methanol and water catalyzed by PtRu/C at the anode (*38*). This capability to operate on high-concentration methanol solutions brings a great advantage for portable applications, such as power sources for motor vehicles and drones due to increased energy density.

Air-breathing DMFCs are appealing due to their simplified design and elimination of oxygen purification and storage components. The performance of the GBP membrane–based DMFC supplied with air was also investigated, achieving a peak power density of 310 mW cm^−2^ at 250°C. This is slightly lower than with pure oxygen at the cathode due to a slight increase in polarization resistance (figs. S46 and S50, A and B). The DMFC operated with 16 M methanol and oxygen also demonstrated favorable operating stability, with a low voltage decay rate of 0.42 mV hour^−1^ at 250°C and 400 mA cm^−2^, owing to the membrane’s excellent thermal and dimensional stability (Fig. 4G). Compared to other advanced DMFCs, the GBP membrane–based DMFC achieves a power density 1.4 times higher than the highest previously reported value (PBI/SiO_2_/PA) (Fig. 4H). This exceptional performance results from greatly reduced methanol crossover and improved proton conductivity at elevated temperatures. The fabrication process of the GBP membranes is also intrinsically scalable. Monolayer PEI-graphene and PEI-BN nanosheets are produced through our previously reported sticky ball-milling method, enabling high-yield exfoliation suitable for gram-scale production. Membrane assembly via vacuum-assisted filtration is readily scalable, and we have fabricated freestanding GB membranes with diameters of ~4 cm using a larger filtration setup (fig. S51). PA loading is performed via solution impregnation, a straightforward and scalable method compatible with large-area membranes. These features collectively highlight the feasibility of scaling GBP membranes for practical high-temperature fuel-cell applications.

## DISCUSSION

In summary, we developed a high-temperature proton-conducting membrane based on 2D protonic nanosheets, using PA to bridge individual nanosheets. Within the 2D nanochannels, PA aligns into layered structures, forming dense hydrogen bond networks that enable ultrafast synergistic proton conduction via both through-nanosheet transport and proton hopping across confined PA. This nanoconfined architecture not only enhances intersheet proton transport but also suppresses PA loss at elevated temperatures. The resulting GBP membrane exhibits outstanding anhydrous proton conductivity (166 mS cm^−1^ at 250°C) and delivers high power densities in hydrogen fuel cells and DMFCs (1011 and 502 mW cm^−2^, respectively), with stable long-term operation. These findings position the GBP membrane as a promising alternative for high-temperature electrochemical devices, addressing the performance limitations of conventional proton-conducting membranes. More broadly, this work opens pathways for designing proton-conducting membranes with high proton conductivity and thermal stability by integrating protonic nanosheets with nanoconfined proton conductors.

## MATERIALS AND METHODS

### Chemicals

PEI (average molecular weight, ~25,000 by LS, branched), graphite (powder, <45 μm, >99.99%), hexagonal-boron nitride (powder, ~1 μm, 98%), PA (85 wt % in H_2_O), platinum on graphitized carbon (Pt/C, 40 wt %), platinum-ruthenium on graphitized carbon (PtRu/C, 50 wt %), polytetrafluoroethylene dispersion (60 wt % in H_2_O), and isopropanol (IPA) were purchased from Sigma-Aldrich and used without further purification. m-PBI powder (0.8 IV) was obtained from PBI Performance Products Inc. and used as received.

### Synthesis of monolayer nanosheets

The monolayer PEI-graphene nanosheets were prepared by a sticky milling method developed by our group (*29*). A viscose PEI was used as a modifier to aid the exfoliation process. In this method, 0.2 g of graphite powder was mixed with 0.8 g of PEI in a 100 ml of ZrO_2_ jar, along with three different sizes of ZrO_2_ balls, including 40 g Φ 10 mm, 80 g Φ 5 mm, and 8 g Φ 0.1 mm. The exfoliation was performed using a planetary ball mill (ZQM-0.4 L, Changsha Mitr Instrument Equipment Co. Ltd.) with a rotation speed of 500 rpm for 10 hours. To disperse the exfoliated nanosheets in water, 40 ml of deionized (DI) water was loaded into the jar, and the mixture was milled at 300 rpm for 30 min. The resulting nanosheets were then washed with a large amount of DI water by vacuum filtration on a Nylon membrane filter (with 0.45-μm pore size and 47-mm diameter) to remove excess PEI. The washed monolayer PEI-graphene nanosheets were redispersed in water through a 30-min ultrasonication. The final product was obtained from the supernatant after a 30-min centrifugation at 3000 rpm to remove unexfoliated thick graphene plates.

The production of monolayer PEI-BN nanosheets followed a similar method to that used for the monolayer PEI-graphene nanosheets, with a few differences in the processing parameters. In this case, a weight ratio of 1:2 of the pristine BN powder to PEI was applied. The main exfoliation process was conducted at a rotation speed of 600 rpm for 15 hours. The subsequent purification process was the same as that used for the fabrication of monolayer PEI-graphene nanosheets.

### Membrane fabrication

The PEI-GB membrane was produced through a two-step vacuum filtration process. PEI-graphene and PEI-BN nanosheet powders were dispersed in DI water to produce nanosheet dispersions at 0.5 mg ml^−1^, respectively. Specific volumes of each dispersion were filtrated onto a Nylon membrane filter (with 0.2-μm pore size and 25-mm diameter) successively to fabricate the PEI-GB membranes with different layer thicknesses. The BN-layer thickness was altered from 3 to 80 μm while keeping a constant 41-μm-thick graphene layer. Additionally, the graphene layer thickness was tuned from 15 to 93 μm with a constant 9-μm-thick BN layer. After fully drying under ambient conditions, the resulting membranes were carefully peeled from the polymer substrate.

### PA doping

The PEI-GB membranes and *m*-PBI membrane were immersed in the PA for a specific duration ranging from 0.5 to 24 hours, leading to varying levels of acid incorporation. After being taken out of the acid, excess PA was gently wiped away with filter paper. After drying overnight at 80°C, the weight of the PA-doped membrane was measured and recorded as $W_{i}$. The loading of PA was herein calculated using the formula

$$\text{PA}\;\text{loading}\;\left(\text{wt}\%\right)=\frac{W_{i}-W_{0}}{W_{i}}\times 100\%$$ (1)

where $W_{0}$ is the original weight of the membrane.

### Characterization of materials and membranes

Transmission electron microscopy (TEM), SAED, and HR-TEM were obtained using a FEI Tecnai G2 T20 operating at an accelerating voltage of 200 kV. AFM was performed in tapping mode using a Bruker Dimension Icon with samples deposited on a cleaned mica substrate. FTIR spectroscopy was conducted from 4000 to 400 cm^−1^ at a resolution of 2 cm^−1^ using a PerkinElmer Spectrum 2 FTIR. TGA was carried out on a TA Instruments SDT 650 with a heating rate of 10°C min^−1^ in air. All samples were preheated at 100°C for 3 hours to remove moisture. Weight loss under isothermal conditions was recorded at 250°C in the air following a preheating process with a heating rate of 1°C min^−1^. Scanning electron microscopy (SEM) and EDX were performed using an FEI Quanta 3D with an operating voltage of 10 kV and a spot size of 3. XPS was acquired using a Thermo Scientific Nexsa Surface Analysis System with an Al K_α_ incident radiation and a hemispherical analyzer. XRD was performed using a Bruker D2 Phaser with a Cu K_α_ radiation source (30 kV and 10 mA) and a 0.1-mm divergence slit at a step size of 0.02°. Solid-state ^31^P NMR spectra of the PA-incorporated membranes were obtained on a Bruker Avance 300-MHz wide-bore spectrometer with a 4-mm cross-polarization magic angle spinning probe using a ZrO_2_ rotor. The spinning rate was 2 kHz, and chemical shifts were referenced relative to 85% H_3_PO_4_ at 0 parts per million.

### Gas permeation tests

The gas permeances of the membranes with various gases were measured at room temperature and 1-bar feed pressure using a custom-built permeation apparatus based on the constant-volume/variable-pressure method, following our previous work (*39*). The flat membrane was secured onto a porous stainless-steel sample holder using epoxy resin (Varian Torr Seal). The holder was then placed inside a Pyrex tube with a flowing feed gas, while its outlet was connected to a pressure transducer (MKS 628B Baratron) and a vacuum pump. The gas permeance of gas i ($P_{i}$) was calculated from the steady-state slope of the downstream pressure versus time ($\frac{\mathrm{dP}}{\mathrm{dt}}$) curve as

$$P_{i}=\frac{\mathrm{VL}}{\mathrm{ART}∆P}\frac{\mathrm{dP}}{\mathrm{dt}}$$ (2)

where V is the volume of the downstream reservoir, L is the membrane thickness, A is the effective membrane area, R is the ideal gas constant, T is the absolute test temperature, ∆P is the transmembrane pressure difference.

### Proton conductivity measurement

The through-plane proton conductivity of the GBP membranes was determined by a two-probe ac impedance method, using a setup similar to the fuel cell testing (fig. S52). The membrane was placed between two carbon papers and clamped with two polar plates. The electrochemical impedance spectroscopy was measured at 0.4 V with a frequency range from 1 MHz to 0.1 Hz using a Gamry Interface 5000E potentiostat without humidification. The through-plane proton conductivity (σ, in millisiemens per centimeter) was then calculated using the following equation

$$\sigma =\frac{L}{R\times A}$$ (3)

where L is the membrane thickness, R is the ohmic resistance obtained from the Nyquist plots (fig. S20), and A is the effective membrane area.

The in-plane proton conductivity was measured via a four-probe ac impedance method (fig. S53). The membrane was cut into a size of 30 mm by 5 mm and placed in contact with four silver electrodes. The in-plane proton conductivity was similarly figured out using Eq. 3, where *L* represents the fixed distance between four probes, *R* is the measured membrane resistance, and *S* is the cross-sectional area of the membrane.

### Electrical conductivity measurement

The through-pane electrical conductivity of the GBP membrane was measured using a homemade apparatus (fig. S54). The membrane was placed between two conductive carbon papers (AvCarb MGL 190) and held together by two glass slides. The two sides of the membrane were connected to a multimeter via conductive copper tape (AT526, RS Components Pty Ltd.). The electrical conductivity was acquired using Eq. 3 with the resistance measured from a multimeter, similar to the calculation of proton conductivity.

### Single-cell performance test

The H_2_-O_2_ fuel cell performance of the GBP and m-PBI/PA membranes was evaluated using a single cell with an effective area of 1 cm^2^. The membrane electrode assembly (MEA) was fabricated using a catalyst-coated substrate method. The catalyst ink was produced by mixing Pt/C (40 wt %) and PTFE emulsion (60 wt %) in IPA/water (3:2 by weight) through 30-min ultrasonication and 20-min probe sonication to obtain a homogeneous dispersion. The cathode and anode gas diffusion electrodes were produced by spray-coating the catalyst ink onto the gas diffusion layer (H24C×483, Freudenberg) to achieve a Pt loading of ~0.7 mg cm^−2^. The electrodes were then heat-treated at 350°C for 2 hours in an Ar atmosphere in a tube furnace (MTI GSL-1100X). During the cell assembly, the prepared membrane was sandwiched between two catalyst-coated papers and fixed with two polyimide films (fig. S52A). The hydrogen fuel cell performance was tested with dry H_2_ (40 ml min^−1^) and O_2_ (40 ml min^−1^) under ambient pressure on the anode and cathode side respectively (fig. S52B). The polarization curves were recorded at different temperatures with a step size of 0.05 V from 1 to 0.2 V.

The DMFC performance of the GBP membrane was assessed using a setup similar to that of the hydrogen fuel cell (fig. S55). The anode used PtRu/C (50 wt %) with a catalyst loading of 1.2 mg cm^−2^, while the cathode remained unchanged from the hydrogen fuel cell. Methanol/water solutions with varying methanol concentrations (5, 10, 16, and 20 M) were injected into the anode via a peristaltic pump operating at a flow rate of 1 ml min^−1^, while oxygen or air was fed into the cathode at a flow rate of 40 ml min^−1^ without back pressure or humidification.

### Methanol crossover measurement

Methanol crossover in the DMFCs was estimated under various temperatures and methanol concentrations using the limit current method. Methanol solution (1 ml min^−1^) and dry nitrogen gas (40 ml min^−1^) were supplied to the anode and cathode respectively. Linear sweep voltammetry was conducted with a scanning rate of 0.005 V s^−1^ from 0 to 1 V.

### MD simulation of PA thermal stability

To validate the retention of PA and water in a confined space, two systems were built and simulated, as shown in fig. S12. For the first system, the slit structure was formed by graphene sheets with an intersheet distance of 1.5 nm and an intersheet gap of 1.2 nm. PA and water molecules (with a mass ratio of 85:15) were filled inside the slit structure. For the box structure, the inner graphene sheets were replaced with PBI monomer molecules (with a total mass matching that of the replaced graphene sheets), and the same numbers of PA and water molecules were filled inside the box structure. The PA, water, and PBI molecules were randomly placed in their systems by using Packmol (*40*). For both systems, the simulation cells are sized with dimensions of 9.24 nm by 1.28 nm by 15.5 nm, all three directions were set as periodic, and the boundaries are indicated by blue lines. To accommodate the possible evaporated water molecules and escaped PA molecules, two vacuum layers with a thickness of 4 nm were left at the bottom and top sides along the *Z* direction. The two systems were first equilibrated with a minimization procedure and then run in an NVT ensemble with a temperature of 300 K and a duration of 10 ns. For the high-temperature cases, the temperature was increased from 300 to 523 K in the first 1 ns under NVT and then kept at 523 K for another 9 ns under NVT. No external electric field was applied in the MD simulations. The simulations are designed to evaluate how nanosheet confinement influences the retention of PA molecules at elevated temperatures, such as 523 K. This setup allows a direct comparison between different structural designs.

### MD simulations of PA confined in 2D channels

The all-atom classic MD simulations were conducted to allow a deepened understanding of the experimental phenomena from an atomistic level, especially for the hydrogen bond networks formed in the nanoconfined 2D channels. The 2D PA mass density distributions were calculated by a code called DensityCalculator (*41*). The Large-scale Atomic/Molecular Massively Parallel Simulator (*42*) was used to perform all MD simulations. The CHARMM General Force Field (*43*) was used to describe the interactions between atoms of all systems, TIP3P water model (*44*) was used to describe the water interactions. The particle-particle particle-mesh algorithm was used to compute the Lennard-Jones and Coulombic interactions with global inner and outer switching cutoffs of 10 Å and 12 Å respectively. Nosé-Hoover thermostat was set for the control of the temperature, and time step was set at 1 fs.

### Hydrogen bond calculations

Because proton transport could be promoted by a stable and strong hydrogen bond network, the characterization of the hydrogen bond network is critical to a better understanding of proton transport. To determine a hydrogen bond, the following distance and angle criteria were applied, see fig. S33 for a detailed illustration

$$r_{\text{AD}}\leq 4Å$$ (4)

α≤90° (5)

To better reflect the strength of one hydrogen bond, we defined a linear distance-dependent weight function, ($r_{\text{cutoff}}-r_{\text{AD}}$)/$r_{\text{cutoff}}$, where $r_{\text{cutoff}}$ is the maximum cutoff length to identify a hydrogen bond and $r_{\text{AD}}$ is the length between the donor and acceptor oxygen atoms. In this study, we set the $r_{\text{cutoff}}$ to 4 Å, a standard value widely used. Both the 1D and 2D hydrogen bond density and strength distributions were calculated by a homemade code, HydrogenBondCalculator (*45*). All hydrogen bond distributions were calculated by averaging the last 1000 frames (the last 1 ns).

### Free proton calculations

A proton is considered in a “free state” when it maintains a distance of at least 1.2 Å from nearby atoms. This threshold accounts for the typical ─OH bond length (0.96 to 1.0 Å) (*46*, *47*), with vibration extending up to 1.2 Å (*48*). At elevated temperatures (e.g., 600 K), the proton-to-nearest atom distance ranges from 0.85 Å to 1.3 Å, centered around ~1.0 Å (fig. S35A), justifying the 1.2 Å cutoff for defining free protons. To assess nanoconfinement effects on proton lifetime and proportion, three systems were chosen: graphene nanosheets confining one, two, and six layers of PA molecules. For each system, we recorded proton states in each frame, setting a proton as “1” if free and “0” otherwise. Continuous “free” segments in a trajectory were counted to calculate the lifetime. For example, if a proton’s states in a trajectory are recorded as “0 0 0 1 1 1 0 0 0 1 0 0 0 0 1 1 0 0 0,” there are three continuous segments of the free proton state: “1 1 1,” “1,” and “1 1.” The lifetime for this proton is calculated as the average of these segment lengths: (3 + 1 + 2)/3 = 2 fs, assuming a time step of 1 fs. This method calculates the lifetime of each proton in the AIMD trajectory, and the average free proton lifetime is then determined by averaging all individual lifetimes. The free proton proportion in each frame is calculated by dividing the number of protons in the free state by the total number of protons in that frame. This approach allows us to track the variation of free proton proportion over time, as shown in fig. S35B. The average free proton proportion for each system is then obtained by averaging these proportions across all frames.

### Single-point energy calculations

The single-point energy calculations of proton transport through monolayer graphene or BN nanosheet were also performed using the Vienna Ab initio Simulation Package (VASP) (*49*), and the Perdew–Burke–Ernzerhof (PBE) exchange-correlation function and the projector-augmented wave (PAW) approach were used. The monolayer graphene or BN nanosheet was placed in the center of a cell with dimensions of 2.02 nm by 1.75 nm by 2.40 nm, and a regular 1 × 1 × 1 mesh centered at Gamma was selected for *k*-points. The proton was set at distances of 1.8 to −0.5 nm from the sheet, and then the relevant energy states were calculated individually. The lowest energy states were found at distances of 1.0 nm to the graphene sheet and 0.9 nm to the BN sheet, while the highest energy states were both at the center of the ring (0 nm). The energy barriers were then calculated as the difference between the highest and lowest values. The rest of the parameters used in the VASP energy barrier calculation are presented in table S7.

For the calculation of energy barriers at different temperatures, we did not directly use the carbon-carbon bond length from the AIMD simulations. Instead, we used the equivalent bond length converted from the ring area, obtained from a homemade code, HexagonalRingCalculator (*50*). In AIMD simulations, some bonds within a ring area are stretched while others are squashed (Fig. 3I). In this case, it is highly improbable for all six bonds in the same ring to be stretched simultaneously. Therefore, the equivalent bond length derived from ring area is a more accurate and direct indicator for energy barrier calculations.

### Arrhenius rate constant calculations

The Arrhenius rate constant is calculated according to

$$k=Ae^{-E/k_{B}T}$$ (6)

where k is the rate constant, A is the preexponential factor, E is the activation energy, or, in this context, the energy barrier, $k_{B}$ is the Boltzmann constant, and T is the temperature. The calculation of the Arrhenius rate constant changes does not require knowing the exact value of A because the changes are represented by a ratio of concerned energy states over the base energy state

$$∆k=\frac{Ae^{-E_{c}/k_{B}T}}{Ae^{-E_{0}/k_{B}T}}=e^{-∆E/k_{B}T}$$ (7)

where ∆k is the rate constant change, $E_{c}$ is the energy barrier of a concerned state, $E_{0}$ is the energy barrier of the base state, and $∆E=E_{c}-E_{0}$ is the energy barrier difference.

For the calculation of the quantum tunneling transformed Arrhenius rate constant, the temperature-dependent preexponential factor and energy barrier were obtained from the literature (*34*).

## Supplementary Materials

### This PDF file includes:

Figs. S1 to S55 Tables S1 to S7 References
