# Supplementary material for: Proton-shuttling nanosheet membranes enable high-power-density protonic fuel cells
Source: Sci Adv. 2026 May 15;12(20):eaea1569. doi: 10.1126/sciadv.aea1569 (PMC13178547; doi:10.1126/sciadv.aea1569)
Supplement: Supplementary file 1 — Figs. S1 to S55 Tables S1 to S7 References [file sciadv.aea1569_sm.pdf]

Science Advances
AAAS

Supplementary Materials for

Proton-shuttling nanosheet membranes enable high-power-density protonic fuel cells

Kaiqiang He *et al.*

Corresponding author: Jefferson Zhe Liu, zhe.liu@unimelb.edu.au; Zongping Shao, zongping.shao@curtin.edu.au; Huanting Wang, huanting.wang@monash.edu

Sci. Adv. **12**, eaea1569 (2026)
DOI: 10.1126/sciadv.aea1569

This PDF file includes:

Figs. S1 to S55
Tables S1 to S7
References

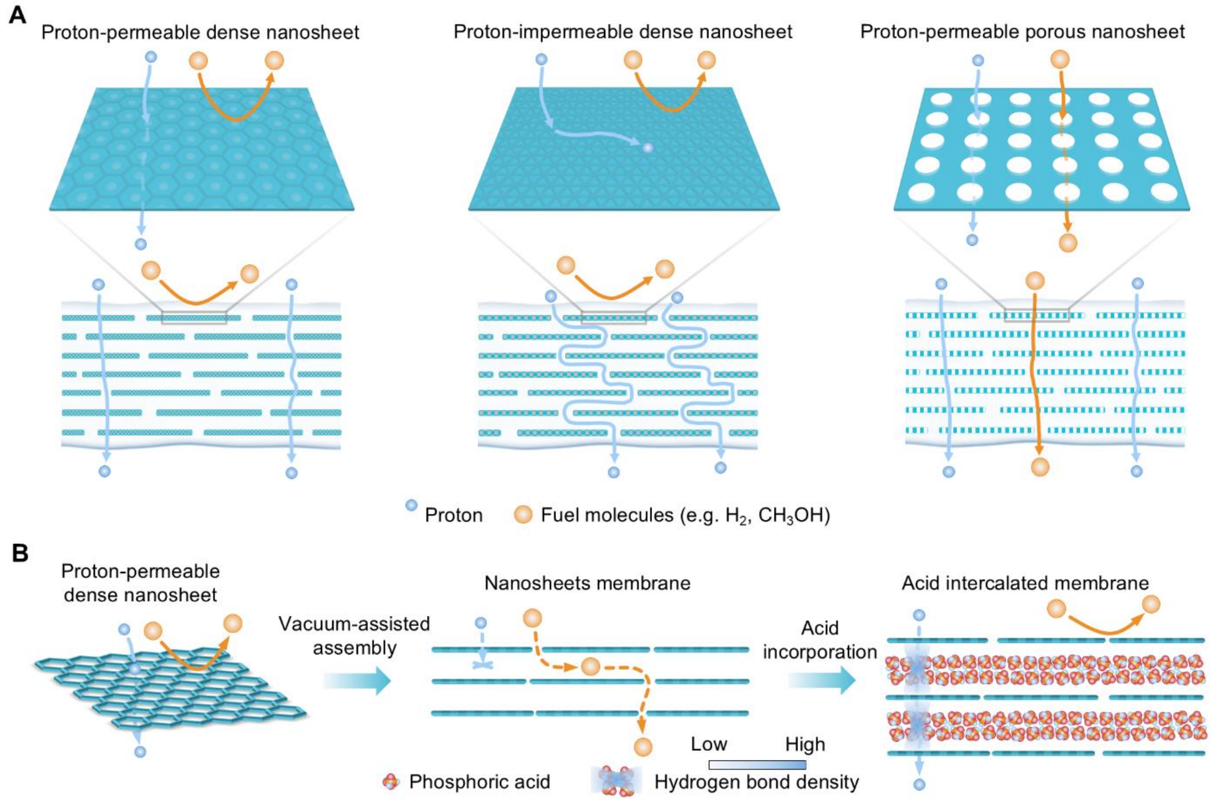

Fig S1. (A) Schematic illustration of proton and fuel molecule translocation through proton-conducting membranes constructed with three types of protonic nanosheets: proton-permeable dense nanosheets (e.g., monolayer graphene and boron nitride nanosheets), proton-impermeable dense nanosheets (e.g., solid acid nanosheets and metal oxide nanosheets), and proton-permeable porous nanosheets (e.g., metal-organic framework and covalent organic framework nanosheets). In proton-permeable dense nanosheets, protons can pass through electron cloud vacancies in hexagonal rings, while fuel molecules are blocked. Proton-impermeable dense nanosheets prevent the passage of both proton and fuel molecules. While protons can migrate along hydrogen-bonded water networks on the surface, stacking nanosheets increases pathway tortuosity and notably impedes through-membrane proton transport. For proton-permeable porous nanosheets, fuel molecules pass easily through pores, and protons are transported via interactions with functional groups on the pore walls. **(B) Schematic illustration of the preparation of proton-conducting membranes using proton-permeable dense nanosheets, highlighting possible transport pathways for protons and fuel molecules.** When assembling proton-permeable dense nanosheets into membranes, proton transport faces high resistance due to the long transport distance within the intersheet spacing and the lattice mismatch between adjacent nanosheets. The nanochannels formed by the stacked sheets also allow some degree of fuel molecule permeation. However, after phosphoric acid is incorporated, the acid molecules become confined within the intersheet spaces, creating a hydrogen bond network that acts as a bridge to facilitate proton transport across the layers. Additionally, the acid-filled nanochannels can effectively block fuel molecule permeation.

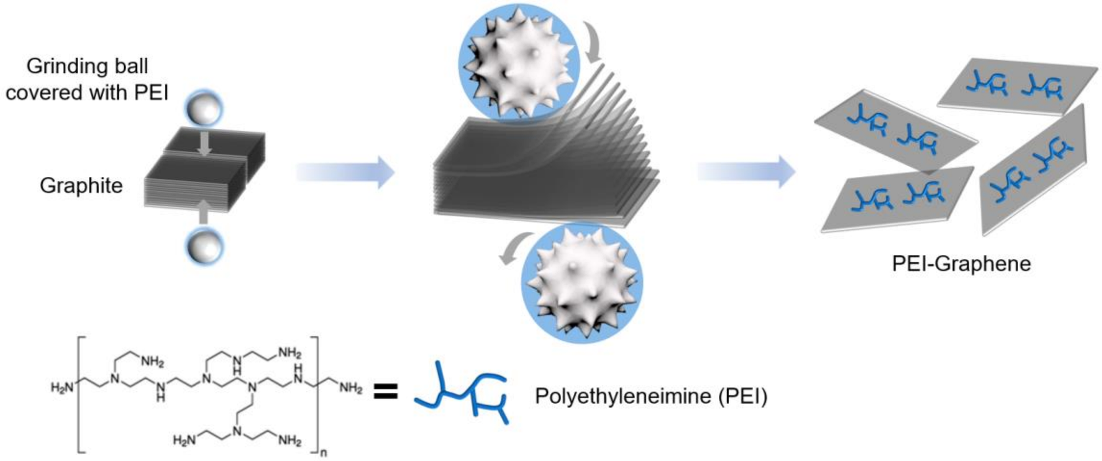

Fig. S2. Schematic depicting the preparation of monolayer PEI-graphene nanosheets via sticky ball milling. During the ball milling process, micrometer-sized ridges on the surface of the grinding balls initially break down the raw materials into smaller pieces. The PEI molecules absorbed on the ball surface function as a buffer layer, reducing the compression forces and preventing excessive breaking. Then, the layered materials undergo delamination due to the strong shear forces generated from the relative sliding of neighboring milling balls with the aid of the sticky polymer.

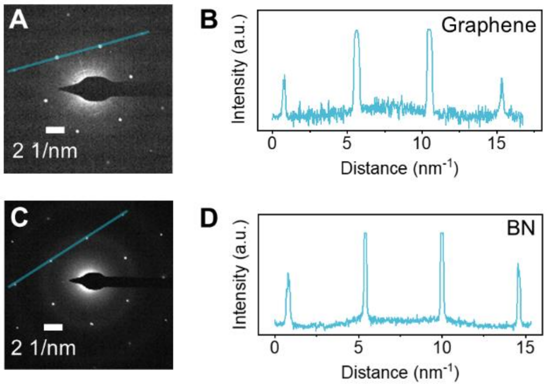

Fig. S3. Selected area electron diffraction (SAED) pattern analysis of the nanosheets. A, SAED pattern of a single-layer graphene nanosheet as shown in Fig. 1C. **B,** Diffraction intensity analysis along the blue line in (A). **C,** SAED pattern of a single-layer BN nanosheet as shown in Fig. 1G. **D,** Diffraction intensity profile along the blue line in (C). In the SAED patterns, the outer and inner spots along the blue lines correspond to {2110} and {1100} facets, respectively. The higher intensity of the inner spots confirms the presence of monolayer nanosheets.

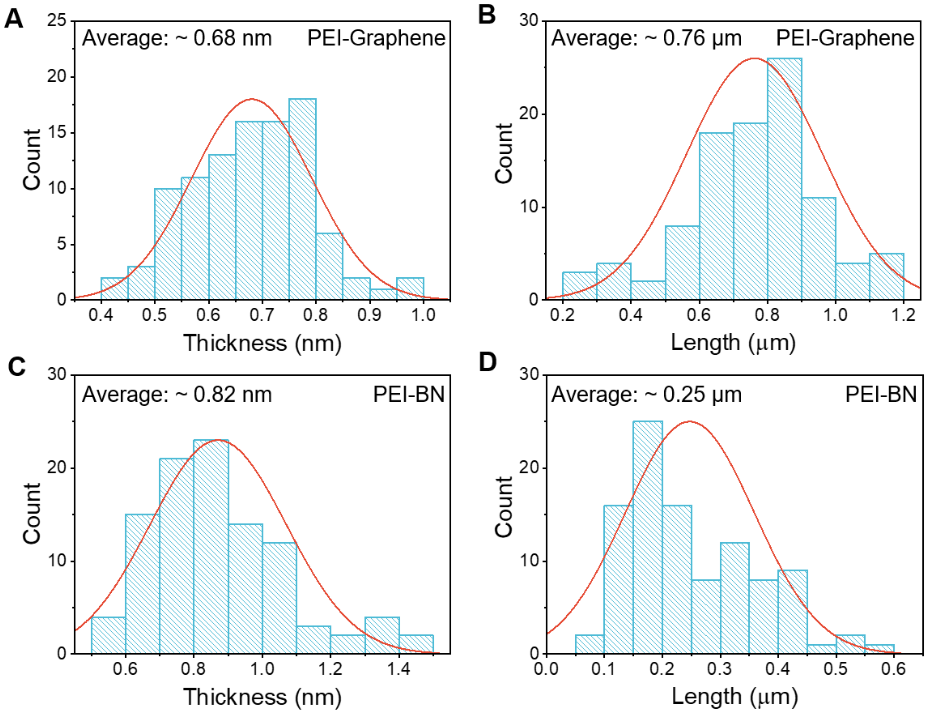

Fig. S4. Analysis of thickness and lateral size distribution of the prepared monolayer PEI-graphene (A and B) and PEI-BN (C and D) nanosheets based on AFM results. The data were obtained from 100 pieces of PEI-graphene and PEI-BN nanosheets respectively.

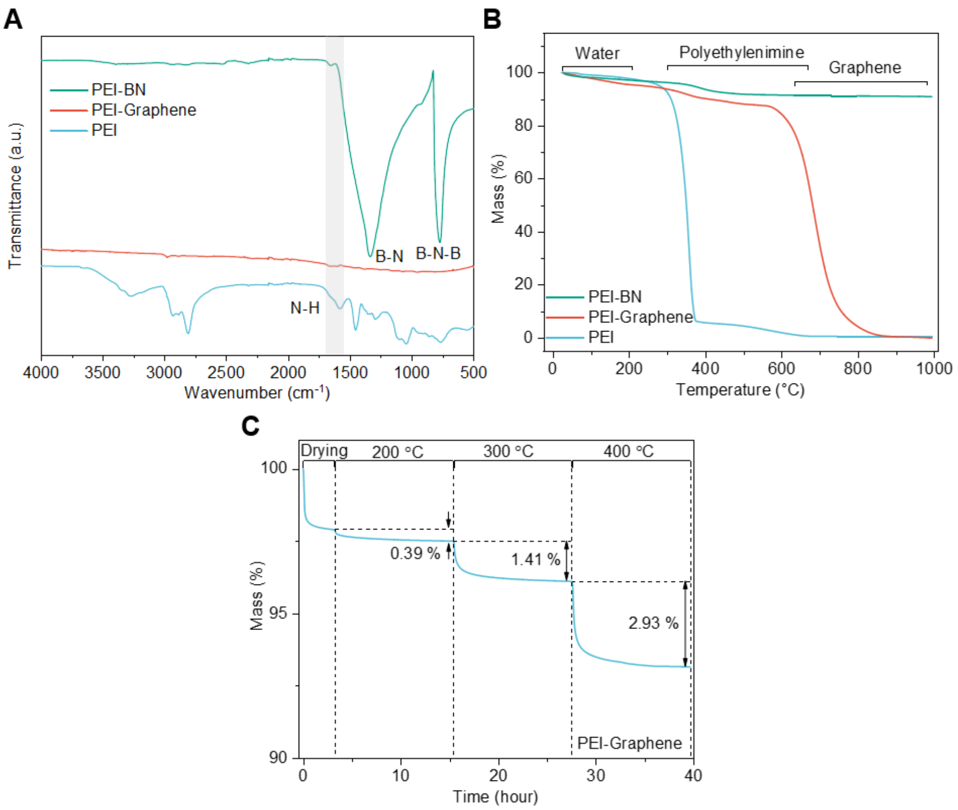

Fig. S5. (A) FTIR spectra of polyethylenimine (PEI), PEI-modified monolayer graphene nanosheet (PEI-Graphene), and PEI-modified monolayer BN nanosheet (PEI-BN). (B) TGA results of PEI, PEI-Graphene, and PEI-BN in air. (C) Isothermal TGA measurements of PEI-Graphene nanosheets in air. The sample was sequentially held at 200, 300, and 400 °C for 12 h each with a heating rate of 10 °C min^{-1}. A pre-treatment at 100 °C for 3 h was performed prior to the measurements to minimize the influence of moisture.

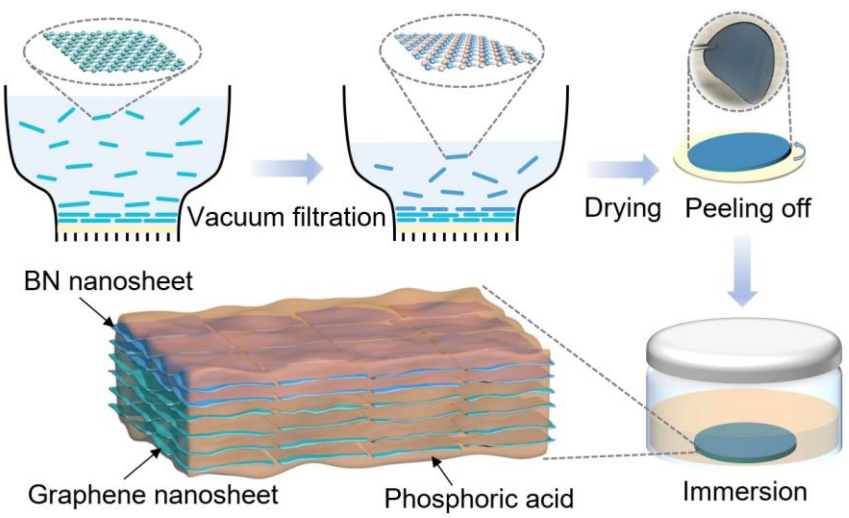

Fig. S6. Schematic illustration showing the fabrication process of the GBP membrane via two-step vacuum filtration and subsequent immersion in PA.

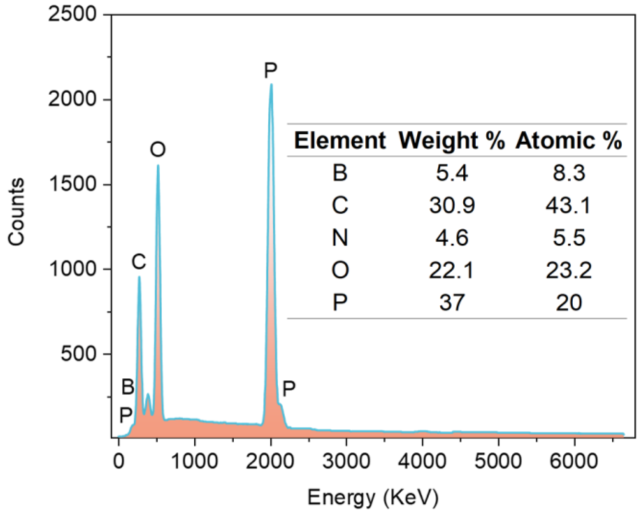

Fig. S7. Elemental composition analysis of the marked area in Fig. 1K using EDX spectroscopy with a relative composition table inserted.

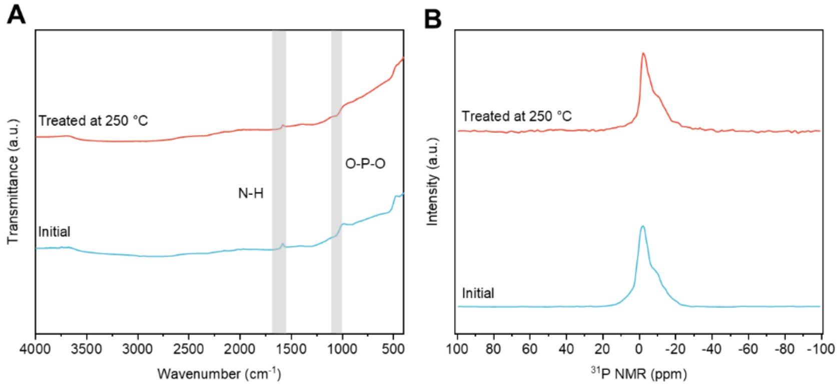

Fig. S8. (A) FTIR and (B) NMR spectra of the GBP membrane before and after being treated at 250 °C in the air for 24 h. The GBP membrane retained its chemical structure, with no noticeable peak shifts or new peaks. This confirms that the observed mass loss is unrelated to structural degradation, validating the thermal stability of the GBP membrane within this temperature range.

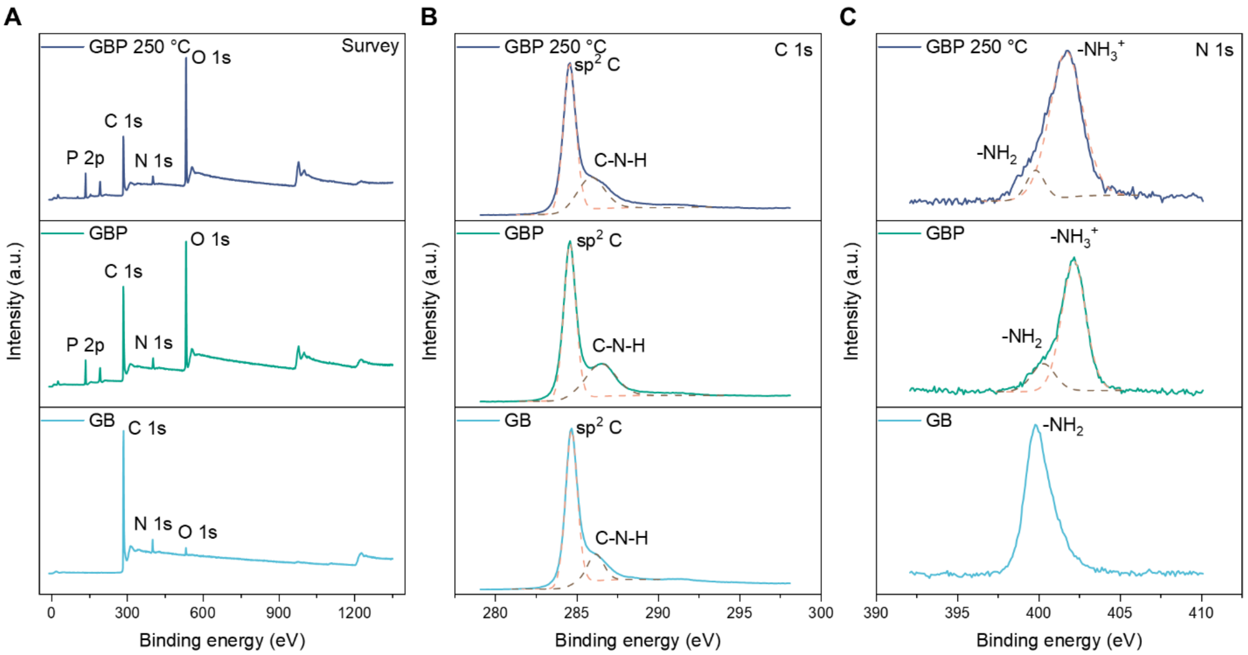

Fig. S9. (A) XPS survey spectra, (B) C 1s and (C) N 1s XPS spectra of the graphene-BN membrane (GB), Graphene/BN/PA membrane (GBP), and GBP membrane treated at 250 °C for 24 h (GBP 250 °C). The examination was conducted on the graphene side. The XPS analysis indicates the presence of carbon, nitrogen, phosphor, and oxygen on the graphene side of the membrane. The high-resolution C1s spectra present two distinct peaks at 284.6 and 286.5 eV, relating to the sp^2-hybridized carbon of graphene and the C-N-H group of PEI. In the N 1s XPS spectra, compared with the peak at 399.8 eV related to the original amine group ($-NH_2$) of PEI, the peak with a higher binding energy of 401.9 eV corresponds to the protonated amine group ($-NH^{3+}$) of PEI, which is surrounded by PA within the GBP membrane. It indicates the grafted PEI molecules on PEI-graphene nanosheets can assist the adsorption of phosphoric acid via electrostatic interaction and hydrogen bonding. The P 2p spectra show a single signature contribution ascribed to P-O of PA (Fig. 2C). Its shift to lower binding energy when the PA is incorporated into the PEI-modified nanosheet membrane, due to the strong chemical interaction between PA and PEI.

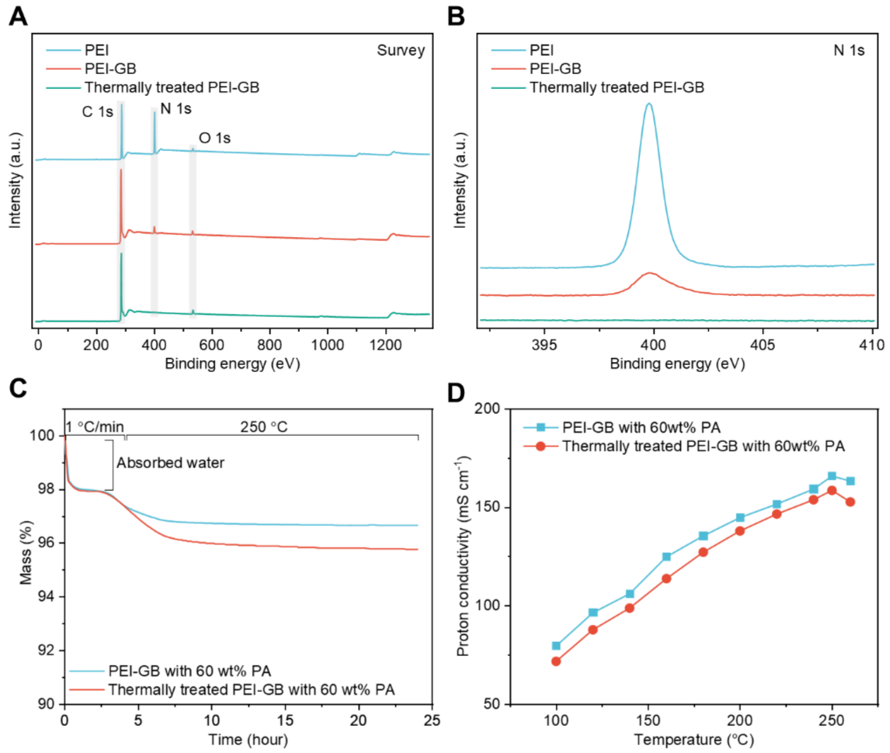

Fig. S10. (A) XPS survey spectra and (B) N 1s XPS spectra of PEI, PEI-GB, and thermally treated PEI-GB membranes. The PEI-GB membrane was thermally treated at 600 °C for 30 min under nitrogen to remove PEI, resulting in an 85% reduction in PEI content, from 8.1 wt% to 1.2 wt%. The XPS tests were conducted on the graphene side. **(C) Recorded mass loss of PEI-GB and thermally treated PEI-GB membranes, both with 60 wt% PA, heated at 1 °C/min to 250 °C and held at 250 °C in air for 24 h. (D) Proton conductivity of the PEI-GB and thermally treated PEI-GB membranes, both incorporated with 60 wt% PA.**

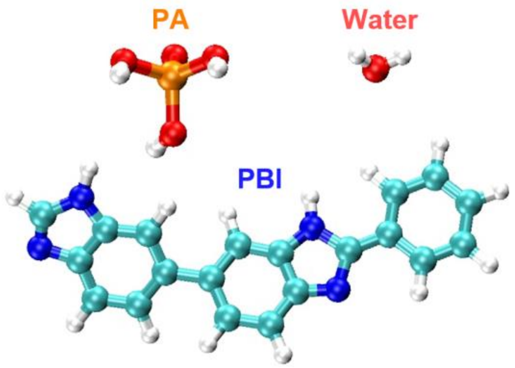

Fig. S11. Chemical structures of phosphoric acid (PA), water, and polybenzimidazole (PBI) used in the MD simulations presented in fig. S12.

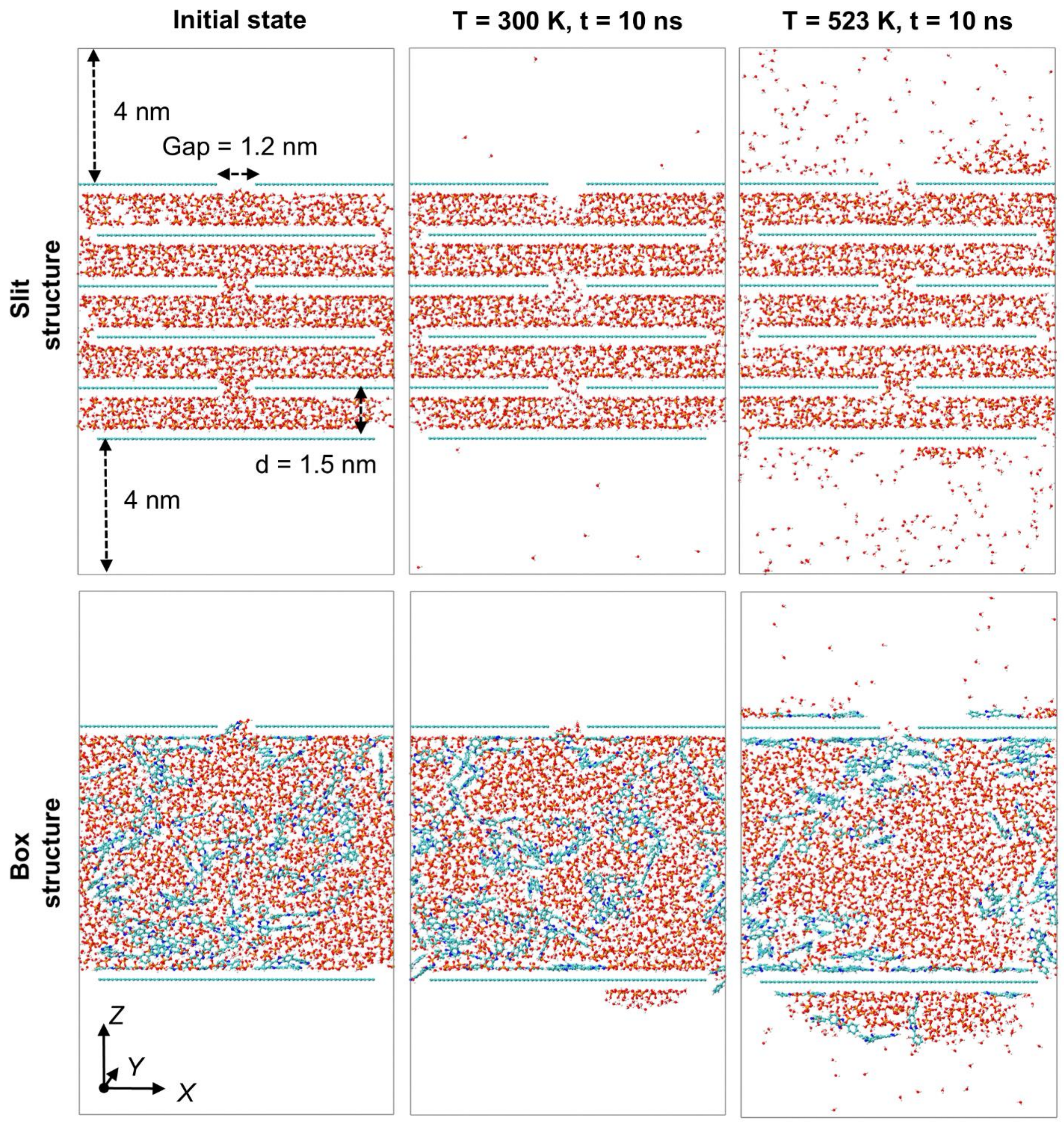

Fig. S12. MD simulation snapshots of the slit and box structures, including their initial states (t=0 ns) and states after 10 ns NVT run at temperatures of 300 K and 523 K.

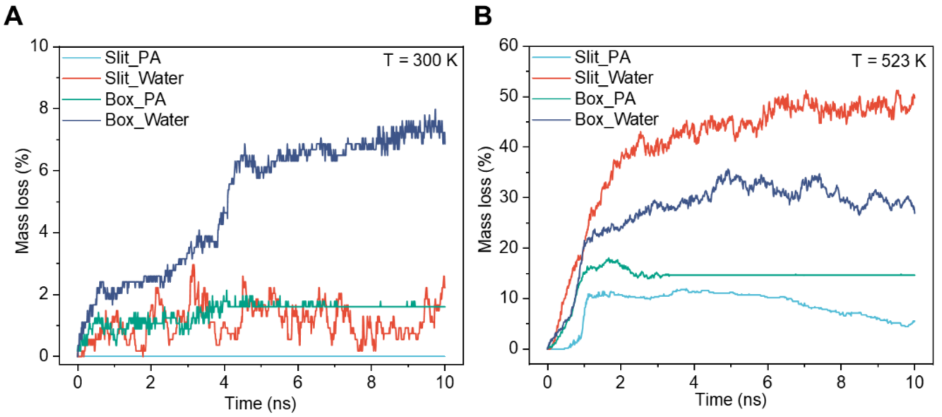

Fig. S13. Comparison of mass change for PA and water in the slit structure and box structure at 300 K (A) and 523 K (B) during 10-ns MD simulation.

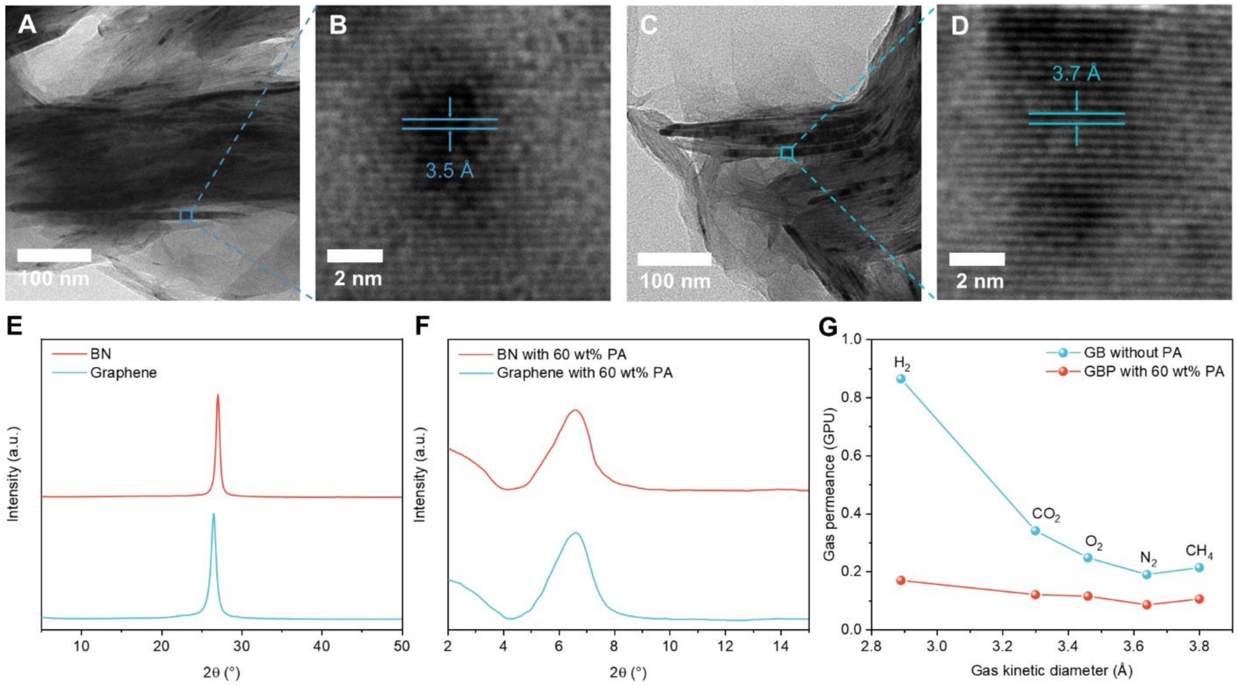

Fig. S14. TEM and corresponding HR-TEM images of the BN layer (A-B) and graphene (C-D) layer from the GB membrane. The TEM samples of the membranes were prepared via ultramicrotomy after embedding them in epoxy. **(E) XRD patterns of the graphene and BN layers from the GB membrane. (F) XRD patterns of the graphene and BN layers from the GBP membrane containing 60 wt% PA. (G) Gas permeation results for the GB membrane (~50 µm, thickness-matched to the GBP membrane) without PA and the GBP membrane with 60 wt% PA at room temperature, using gases with various kinetic diameters.**

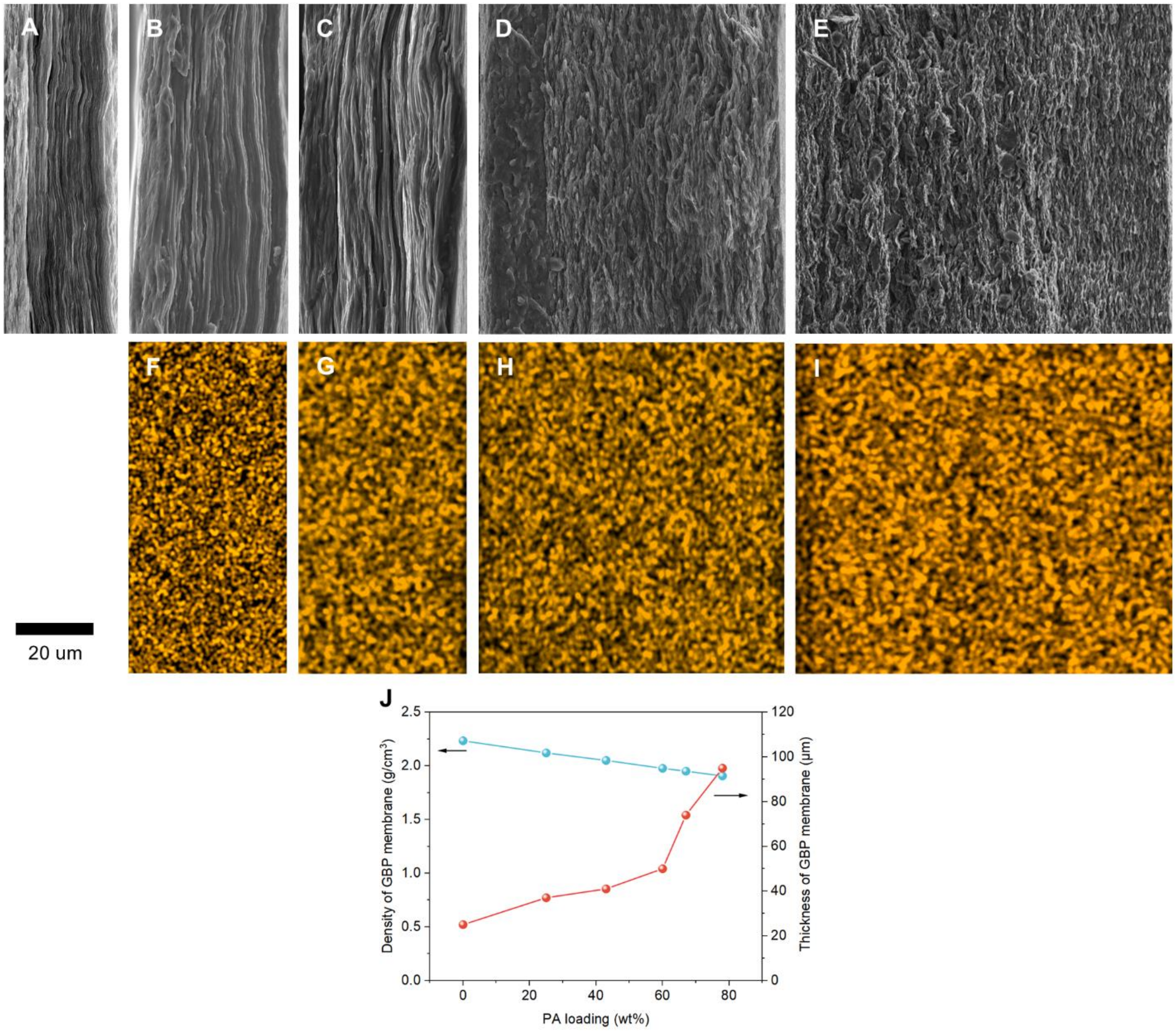

Fig. S15. SEM cross-sectional images (A-E) and corresponding EDX mapping (F-I) of phosphorus of the GBP membranes with different PA loadings (0, 25, 43, 67, and 78 wt%). (J) Changes in density and thickness of the GBP membranes with different PA loading. The densities of the GBP membranes with varying PA loadings were determined by measuring their weight with an electronic balance and their dimensions and thickness with a Vernier caliper and micrometer. For each PA loading level, at least three samples were prepared, and the average value was used for the final calculations.

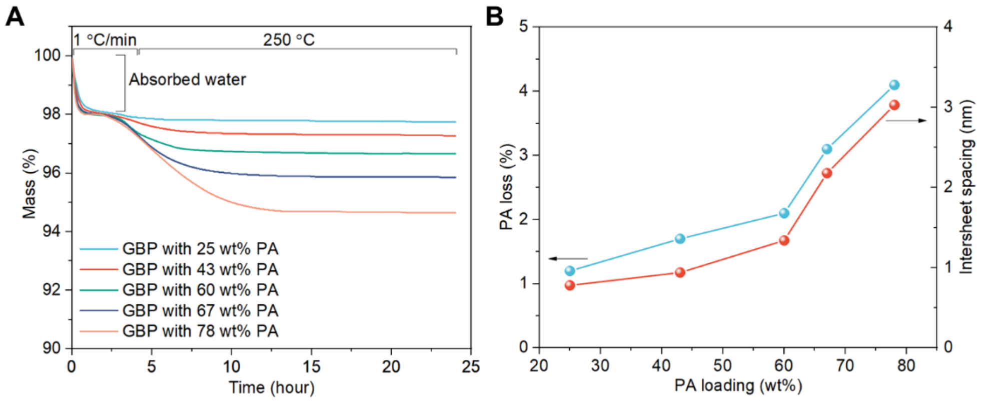

Fig. S16. (A) Recorded mass loss of GBP membranes with different PA loadings when heated at a rate of 1 °C min^{-1} to 250 and kept at 250 in air for 24 h. (B) Change of PA loss from the GBP membranes with different PA loadings obtained from (A). All samples underwent a pre-treatment at 100 °C for 3 h before TGA testing to minimize moisture influence. The absorbed water, including water from the original 85 wt% PA solution, mostly evaporates below 200 °C. The PA loss is estimated from the additional mass loss above 200 °C and during the hold at 250 °C.

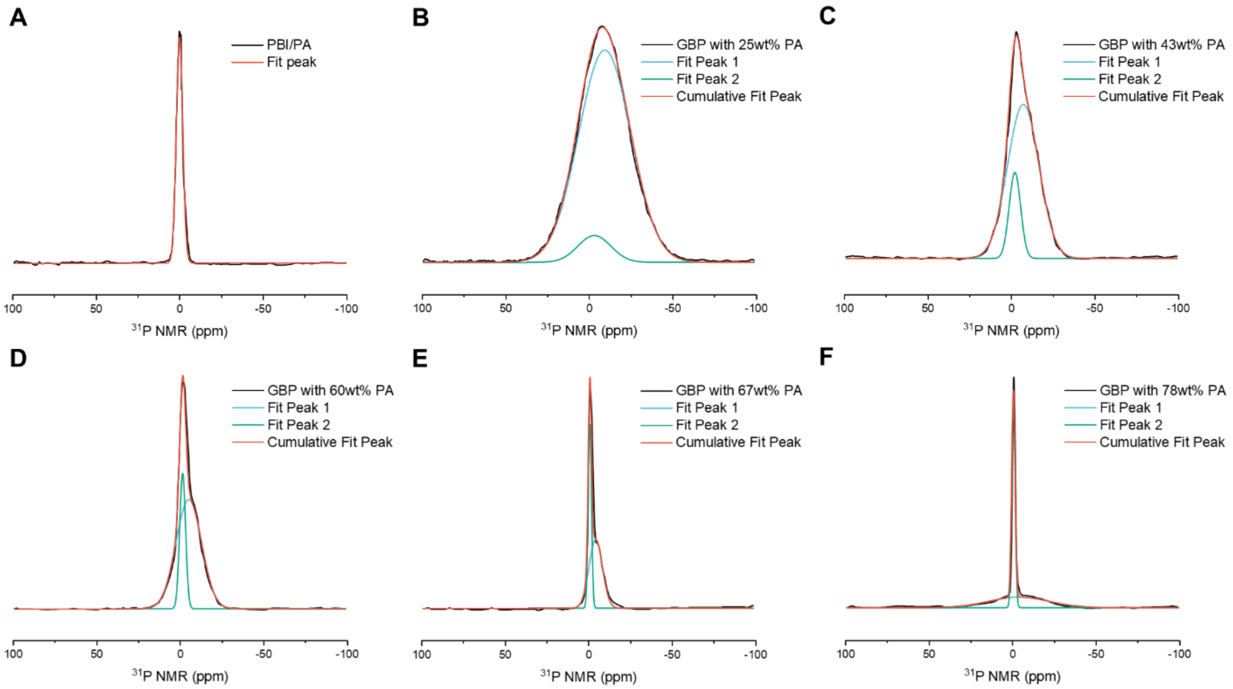

Fig. S17. Solid-state ^{31}P NMR spectra of (A) PA-doped polybenzimidazole membrane (PBI/PA) and (B to F) Graphene/BN/PA (GBP) membranes with 25, 43, 60, 67, and 78 wt% PA incorporated. Each spectrum is fitted with a Gaussian function, where the as-obtained spectrum is shown in black, the fitted peaks are shown in blue and green, and the total fitted curve is shown in red. For the spectra of the GBP membranes, the left peak (in green) represents the weakly absorbed PA molecules in the 2D nanochannels, and the right peak (in blue) relates to the strongly absorbed PA. The solid-state ^{31}P NMR spectra of PBI/PA exhibit a single resonance at 0.04 ppm, indicating PA in a free state. In the case of GBP membranes, two distinct ^{31}P resonance peaks can be observed. The left peak corresponds to weakly absorbed PA within the 2D nanochannels, resembling the bulk state. The right peak represents strongly absorbed PA, influenced by the ring-current effects of the nanosheets and the interactions between PA and PEI molecules (*51–53*).

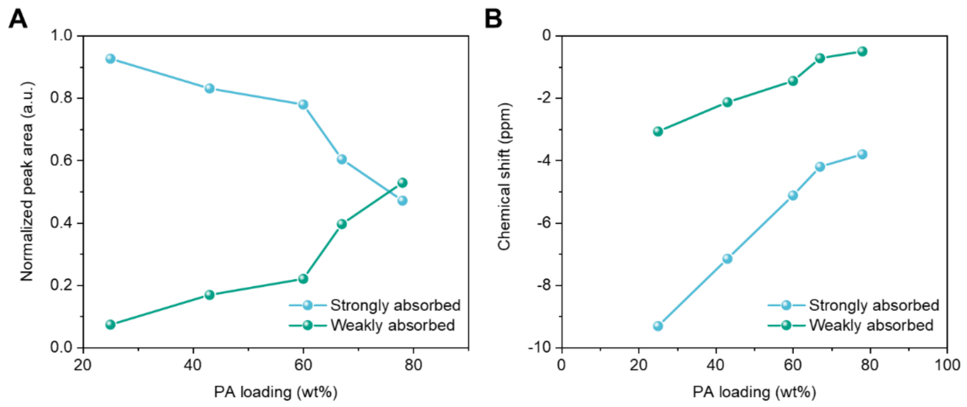

Fig. S18. (A) Normalized peak area, and (B) Chemical shifts of peaks relating to strongly and weakly absorbed PA molecules in the GBP membranes with different PA loading. The data were analysed from fig. S17.

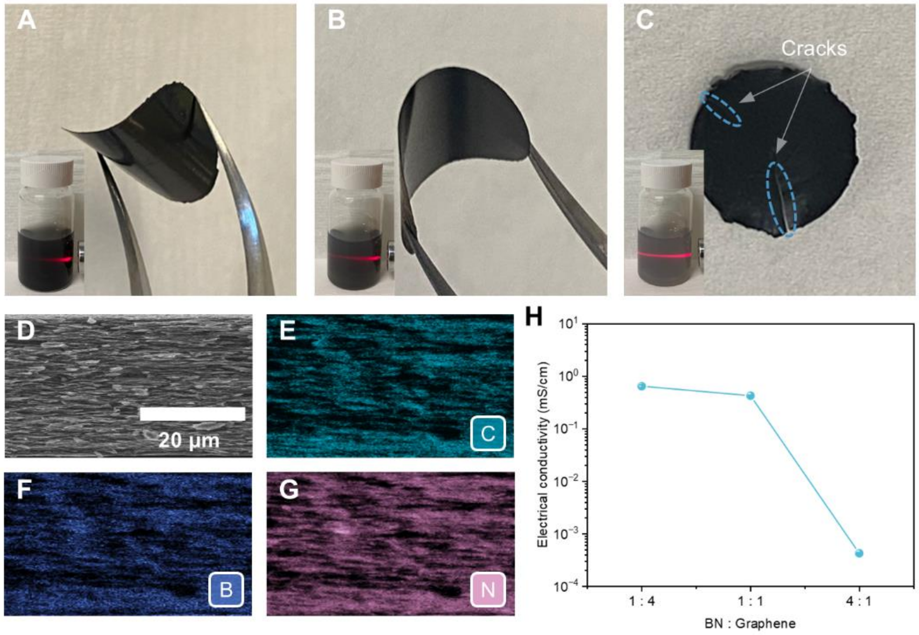

Fig. S19. Characterization of nanosheet membranes with uniformly mixed PEI-graphene and PEI-BN nanosheets. (**A-C**) Photos of uniformly mixed nanosheet membranes with BN to graphene mass ratios of 1:4 (A), 1:1 (B), and 4:1 (C), with insets showing the Tyndall effect in corresponding mixed nanosheet solutions after stirring for 3 h. (**D**) SEM cross-sectional image and EDX maps (**E-G**) of the 1:1 BN-graphene membrane. (**H**) Electrical conductivity of uniformly mixed nanosheet membranes with different BN to graphene ratios, all incorporated with 60 wt% PA.

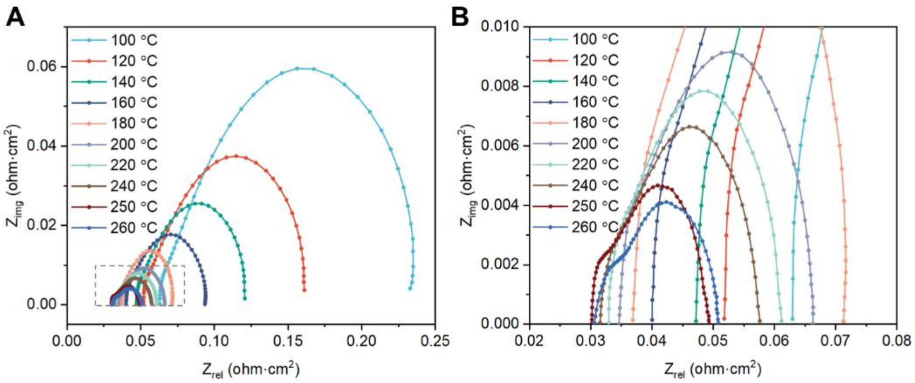

Fig. S20. (A) Nyquist plot of the 50 µm-thick GBP membrane with 60 wt% PA loaded and the corresponding locally magnified plot (B).

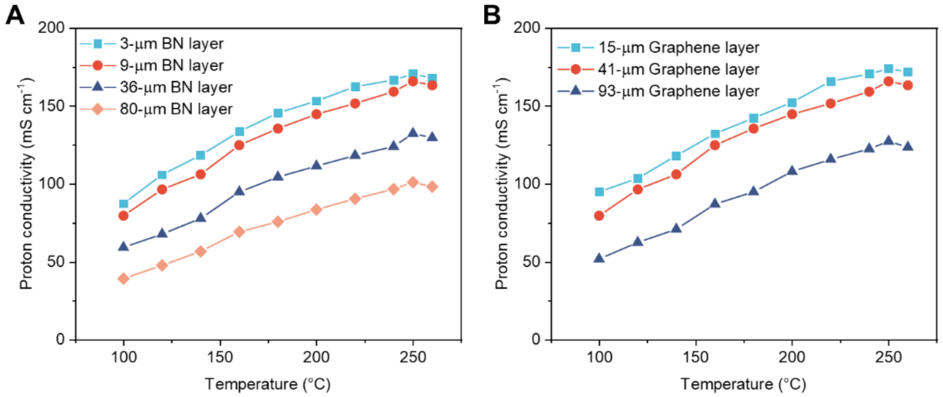

Fig. S21. Temperature-dependent through-plane proton conductivity of 60 wt% PA-doped GBP membranes with 3, 9, 36, and 80 µm-thick BN layers above a constant 41 µm-thick graphene layer (A) and with 15, 41 and 93 µm-thick graphene layers under a constant 9 µm-thick BN layer (B).

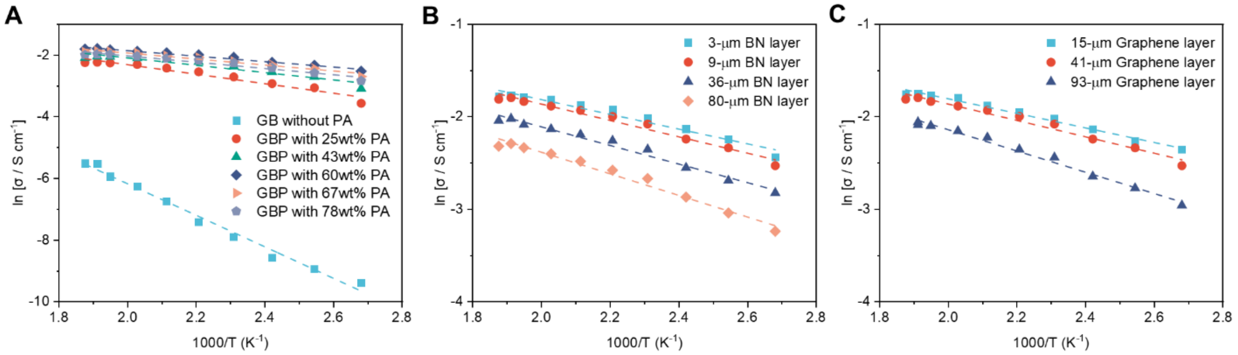

Fig. S22. Arrhenius plots and related linear fitting curves of the proton conductivity of (A) Graphene/BN (GB) membrane and Graphene/BN/PA (GBP) membrane with different PA loading, (B) GBP membranes with various BN-layer thicknesses, and (C) GBP membranes with different graphene-layer thicknesses.

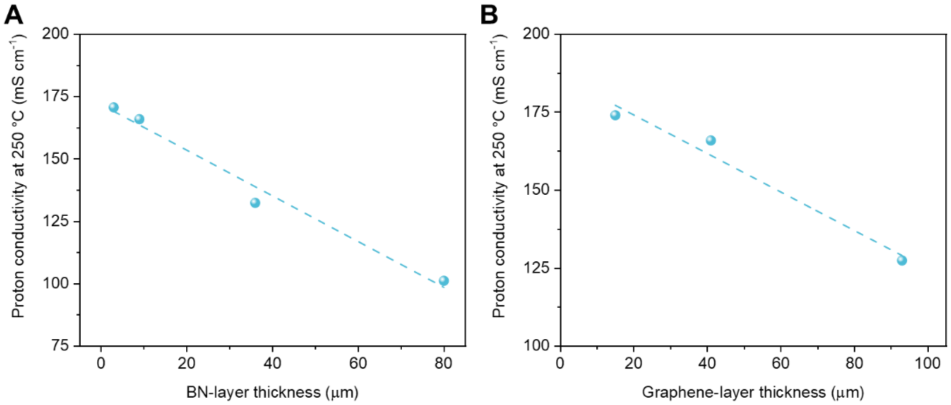

Fig. S23. Proton conductivity at 250 °C varying with BN-layer thickness (A) and graphene-layer thickness (B). A linear correlation between proton conductivity at 250°C and the thickness of the BN or graphene layer is observed. From the Y-axis intercepts in plot (A), the proton conductivity of the pure 41 μm-thick graphene/PA membrane is estimated to be ~172 mS cm^{-1}. Similarly, the proton conductivity of the pure 9 μm-thick BN/PA membrane is estimated to be approximately 186 mS cm^{-1}, as shown in plot (B).

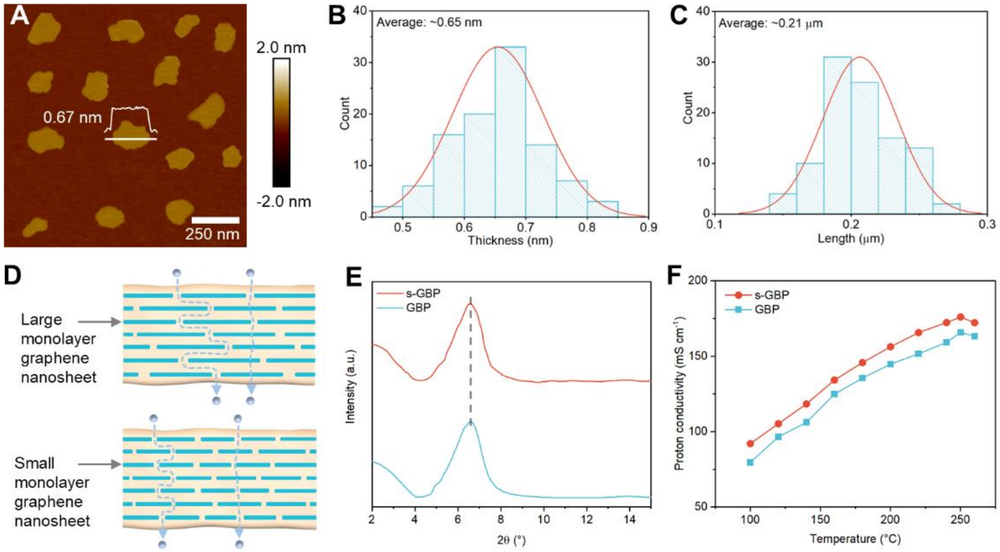

Fig. S24. Effect of PEI-graphene nanosheet lateral size on proton transport in the GBP membrane. (**A**) AFM image of small monolayer PEI-graphene nanosheets with a height profile of a marked nanosheet inserted. (**B**) Thickness and (**C**) lateral size distribution of the small monolayer PEI-graphene nanosheets, based on AFM analysis of 100 nanosheets. (**D**) Schematic illustration of proton transport through large and small monolayer graphene nanosheets stacked with PA. (**E**) XRD curves and (**F**) proton conductivity of the pristine GBP membrane and GBP membrane with small monolayer PEI-graphene nanosheets (s-GBP), both incorporated with 60 wt% PA. The original monolayer PEI-graphene nanosheets, with their higher aspect ratio, provide more tunable geometry compared to the monolayer PEI-BN produced by our sticky ball milling method. As described in our previous work (*29*), the lateral size of monolayer graphene nanosheets can be modified by adjusting the milling time. Longer milling times cause repeated in-plane breakages, resulting in smaller nanosheets. The GBP membrane with smaller monolayer PEI-graphene nanosheets was produced while keeping other fabrication parameters constant.

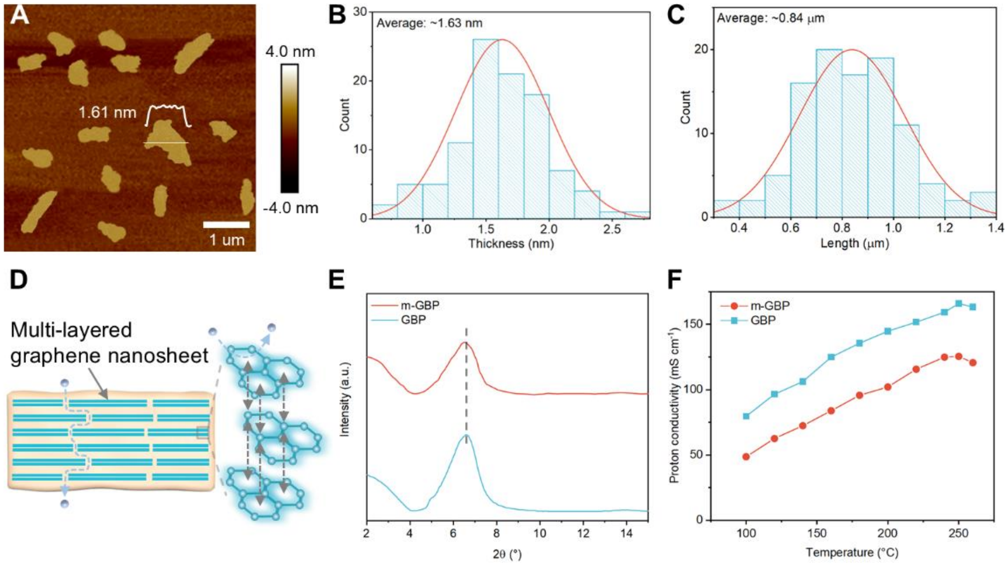

Fig. S25. Effect of PEI-graphene nanosheet thickness on proton transport in the GBP membrane. (**A**) AFM image of multi-layered PEI-graphene nanosheets with a height profile of a marked nanosheet inserted. (**B**) Thickness and (**C**) lateral size distribution of the multi-layered PEI-graphene nanosheets, based on AFM analysis of 100 nanosheets. (**D**) Schematic illustration of proton transport through multi-layered graphene nanosheets stacked with PA. (**E**) XRD curves and (**F**) proton conductivity of the pristine GBP membrane and GBP membrane with multi-layered PEI-graphene nanosheets (m-GBP), both incorporated with 60 wt% PA. The multi-layered PEI-graphene nanosheets were prepared by increasing the PEI-to-graphite ratio from 4:1 to 8:1 in the sticky ball milling process, resulting in inefficient exfoliation, as discussed in our previous work (*29*). The GBP membrane with multi-layered PEI-graphene nanosheets was fabricated with all other parameters kept unchanged.

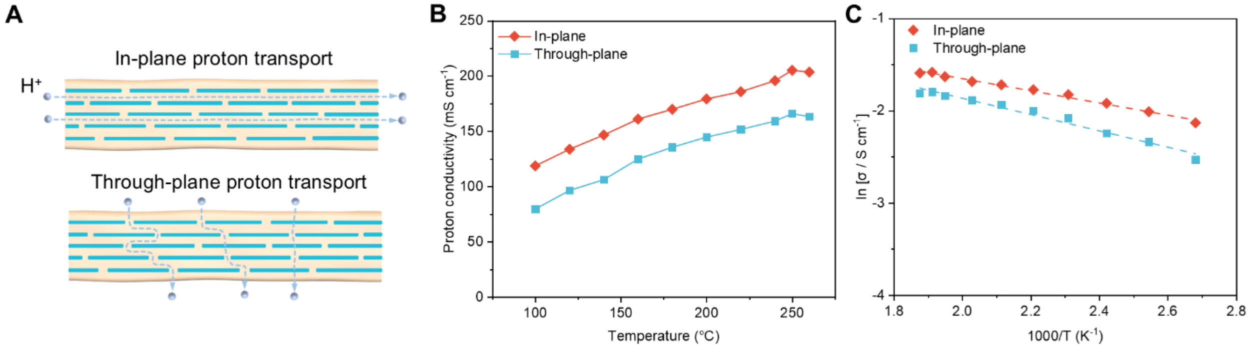

Fig. S26. (A) Diagrams showing the in-/through-plane proton transport pathways within the GBP membrane. Proton conductivity (B), and Arrhenius plots with related linear fitting curves (C) of the in-/through-plane proton conductivity of the GBP membranes with 60 wt% PA and 50-μm thickness. The corresponding activation energies are estimated from the slopes as 5.6 and 7.7 kJ mol^{-1} for the in-plane and through-plane proton transport, respectively. For in-plane proton transport, protons primarily move along the nanochannels within the membrane, with a path tortuosity close to one. Through-plane proton transport combines through-nanosheet and along-nanochannel mechanisms. The nominal path tortuosity for through-plane transport, estimated from the nanosheet length divided by the intersheet spacing of 1.34 nm, is 567 for graphene and 187 for BN layers, respectively.

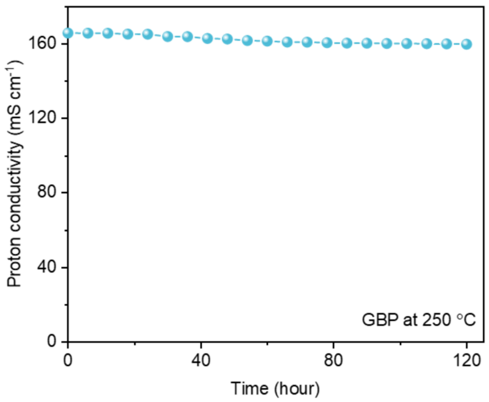

Fig. S27. Recorded proton conductivity changes at 250 °C as a function of time for the GBP membranes with 60 wt% PA and 50-μm thickness.

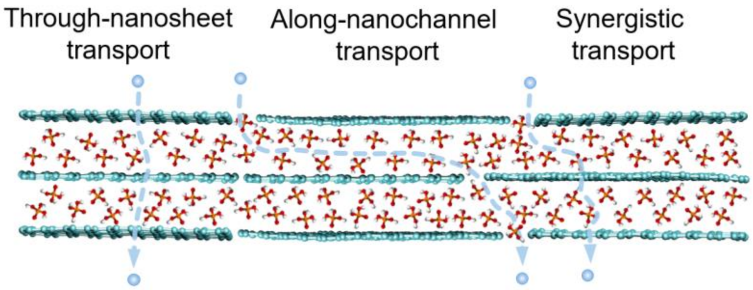

Fig. S28. Schematic showing three possible proton transport pathways through the GBP membrane. In through-nanosheet transport, protons pass through nanosheets via vacancies in the electron cloud and are then transferred through the intersheet spaces to neighboring nanosheets. In along-nanochannel transport, protons hop along the hydrogen bond network formed by the confined PA molecules within the intersheet spacings. At elevated temperatures, enhanced through-nanosheet proton conduction enables a synergistic mechanism that integrates both transport modes. Protons traverse through nanosheets and proceed along the hydrogen-bond network. This combined transport pathway substantially reduces tortuosity, facilitating efficient proton conduction.

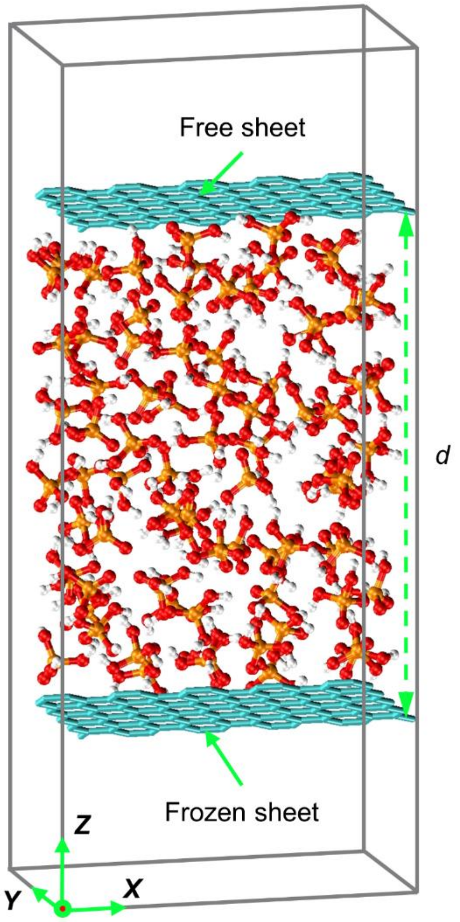

Fig. S29. An example showing the initial configuration of the nanoconfined channel with an intersheet distance of 30 Å. (Note: The simulations focused on the nanosheets without considering the influence of water molecules, as most of the water was evaporated at 250 ℃.) To explore the facilitated proton transport in the nanochannels with confined PA, we built the confined space by using two graphene sheets, between them the PA molecules were randomly placed by using Packmol, and the initial PA mass density was set at 1.88 g cm^{-3}. The simulation cells have the dimensions of 2.23 nm × 1.28 nm × 5.0 nm with the membrane placed at the center, all three directions were set as periodic, and a sufficient vacuum space (⩾ 2.0 nm, depending on cases) was left to avoid the interaction between the graphene sheets across the periodic boundaries, the periodic boundaries are indicated by the blue lines. 23 cases with different initial configurations were built, as listed in table S6. To obtain an equilibrium state of PA molecules in graphene nanoconfined channels, the bottom graphene sheet was frozen after an energy minimization procedure of the whole system, and the upper graphene sheet was left free. Then, the system ran from 300 K to 523 K under the NVT ensemble for 1ns and then ran at 523 K for another 2 ns to collect data for analysis.

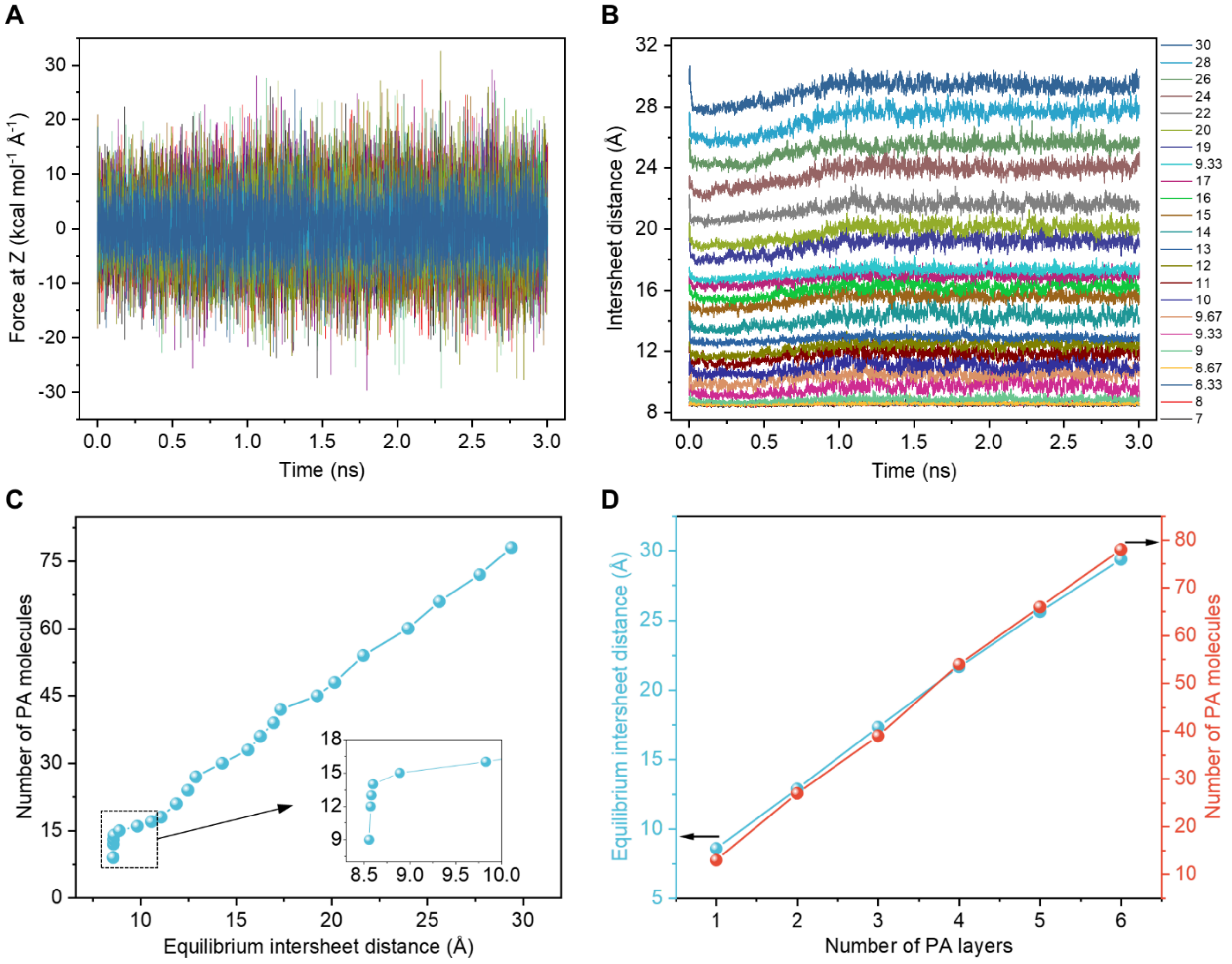

Fig. S30. Fully-equilibrated configuration of the total Z direction force applied on the upper graphene sheet (A), and the intersheet distance between the two graphene sheets (B). (C) Change of the number of PA molecules introduced into the space between two nanosheets as a function of the equilibrium intersheet distance. (D) Variation of equilibrium intersheet distance and the number of PA molecules as functions of PA-layer number. To determine if the equilibrium state is reached, the time-dependent Z-direction total force applied on the upper free graphene sheet and the intersheet distance between the two graphene sheets were recorded and plotted. It can be observed that the forces for all configurations fluctuated around zero with marginal fluctuations, and the profiles of intersheet distance became flat after only 1ns. These two observations indicate the systems are all in an equilibrium state. The equilibrium intersheet distance changed with the number of PA molecules. In the first four configurations (with initial distance from 7 to 8.67 Å), the equilibrium intersheet distances are almost identical, indicating that it is not enough to form a continuous PA layer with these amounts of PA molecules. From an initial intersheet distance of 9 Å, the equilibrium intersheet distance starts to increase with the increasing number of PA molecules, suggesting that the relevant number of PA molecules starts to be more than those in one full PA layer.

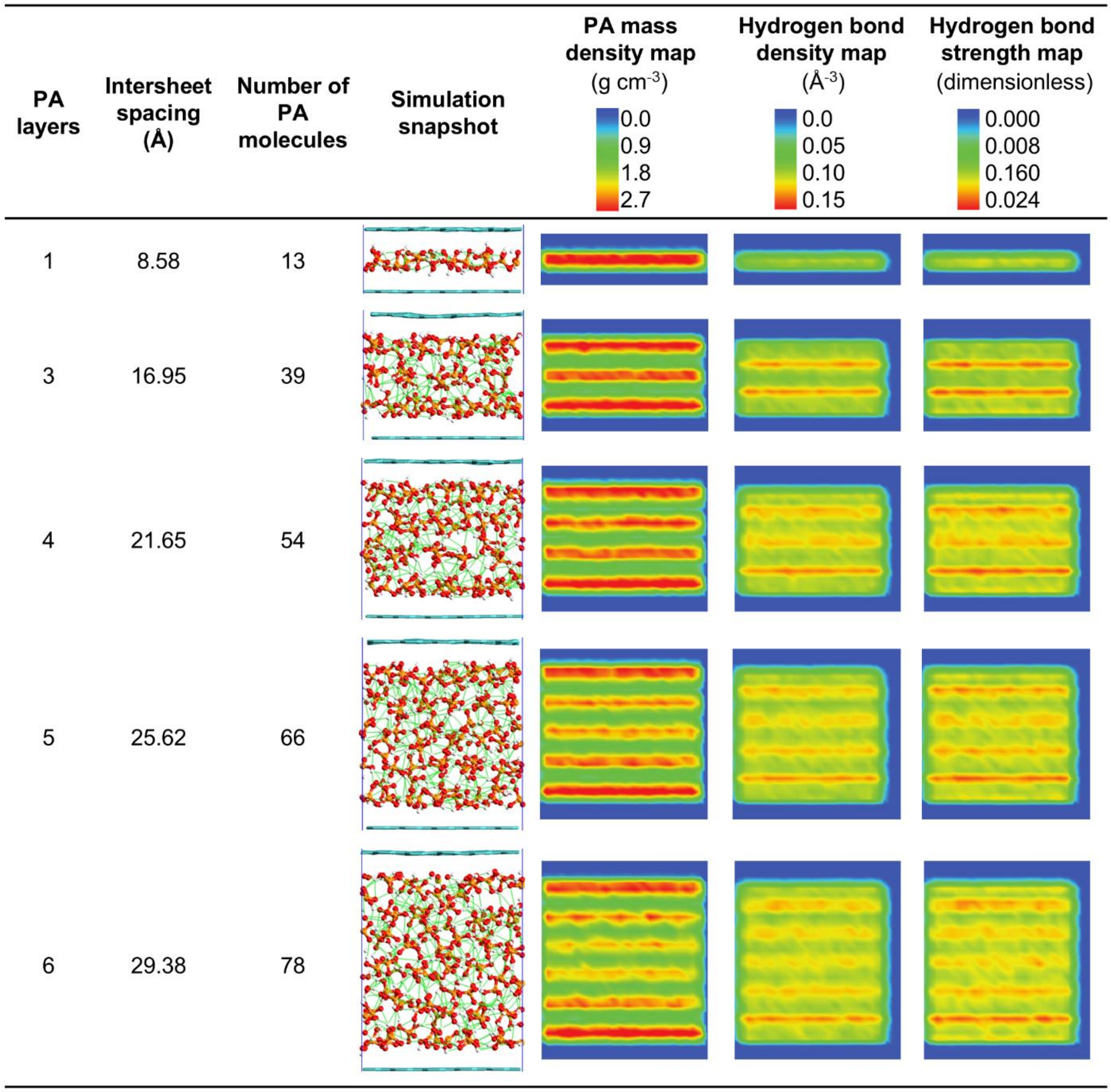

Fig. S31. The simulation snapshots showing the two-dimensional distribution of PA mass density, Hydrogen bond (HB) number density, and HB strength for different amounts of PA molecules confined in the nanochannels with various intersheet spacings formed by graphene nanosheets. The layering phenomenon becomes more evident for PA molecules closer to the graphene sheets. More than two PA layers can be distinguished from the PA mass density maps. The layering effect becomes less apparent with an increase in intersheet spacing (from 0.86 to 2.94 nm). As a single PA molecule can be both the donor and acceptor of multiple HBs, the PA molecule distribution determines the formation and distribution of the HB network. Surprisingly, the HB bond number density and strength distributions correlate well with the mass distributions. Moreover, the HB density and strength peaks counter-intuitively appear at the center of two neighboring mass density peaks. The HB network structure indicates the most active sites for proton hopping are located at the center of two neighboring PA layers. Therefore, it could be inferred that multiple and well-layered PA molecule distribution plays a key role in facilitating and accelerating the proton transport. As the number of PA molecules increases, the equilibrium intersheet distance increases linearly with the number of PA layers (fig. S30D). However, the layered structure of PA becomes less distinct in the middle when the intersheet distance further increases to above 2.17 nm, indicating the reduction of nanoconfined effects.

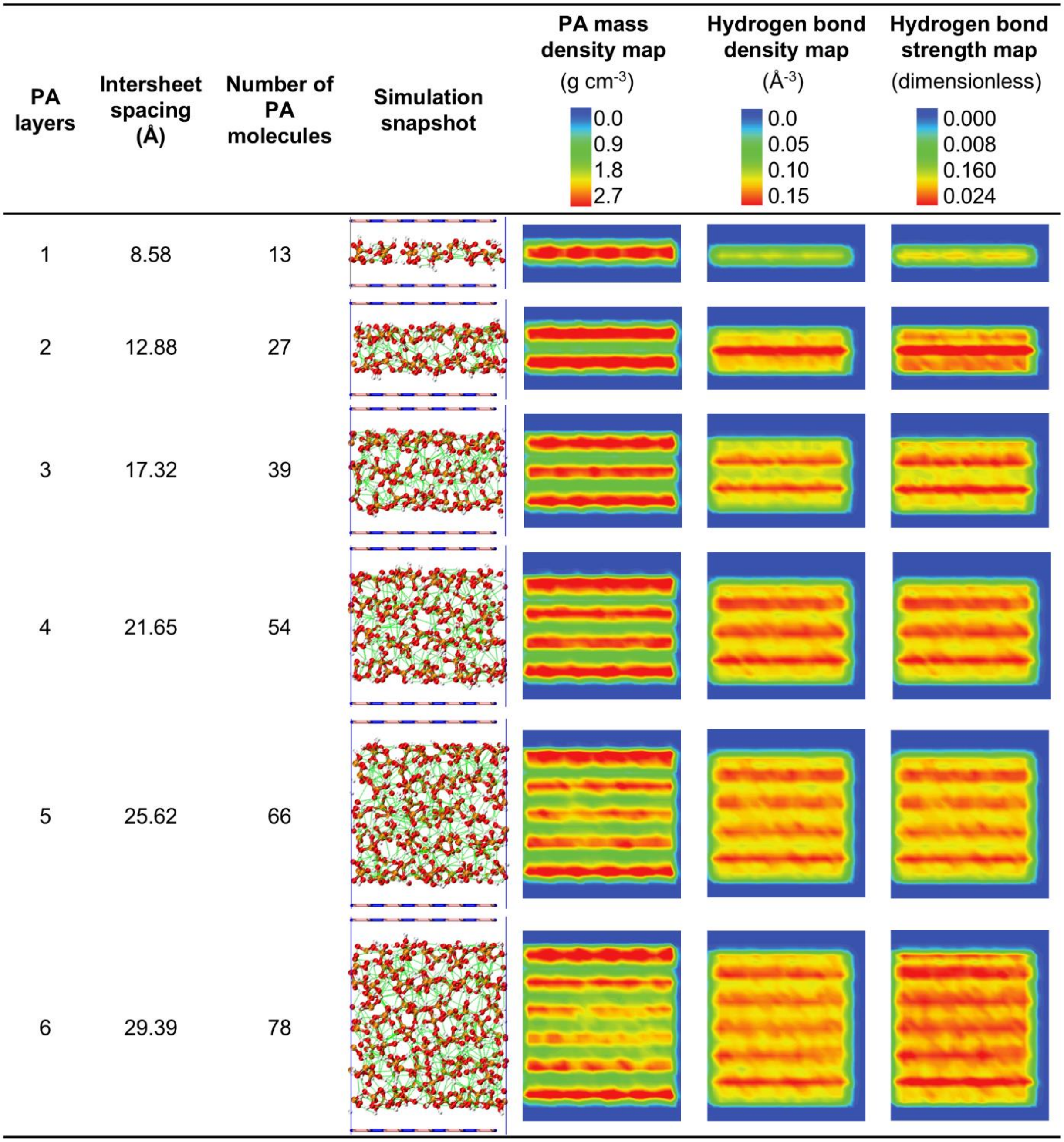

Fig. S32. The simulation snapshots showing the two-dimensional distribution of PA mass density, Hydrogen bond (HB) number density, and HB strength for different amounts of PA molecules confined in the nanochannels with various intersheet spacings formed by BN nanosheets. When forming multiple PA layers, the regions exhibiting the highest hydrogen bond number density and strength are situated in the middle of two adjacent layers, which serve as the most active areas for proton hopping.

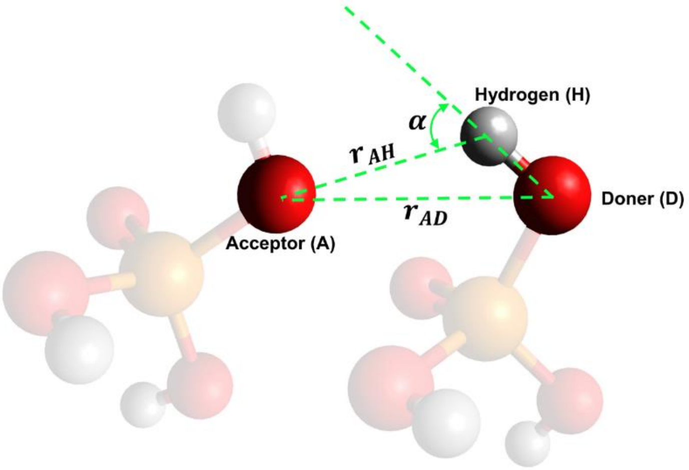

Fig. S33. Schematic illustration of hydrogen bond definition. PA can provide both proton and donor and acceptor sites. In liquid and bulk states, the abundant hydrogen and oxygen in PA molecules enable the formation of a hydrogen bond network through intermolecular hydrogen bonding. This network facilitates a chain-like proton hopping from one PA molecule to another, resulting in a continuous proton flow via the Grotthuss mechanism. Hydrogen bond strength represents the relative length of a hydrogen bond. A shorter hydrogen bond often means a shorter proton hopping pathway and a shorter timescale for proton transport. Therefore, the hydrogen bond strength can be used to indicate the proton transport rate.

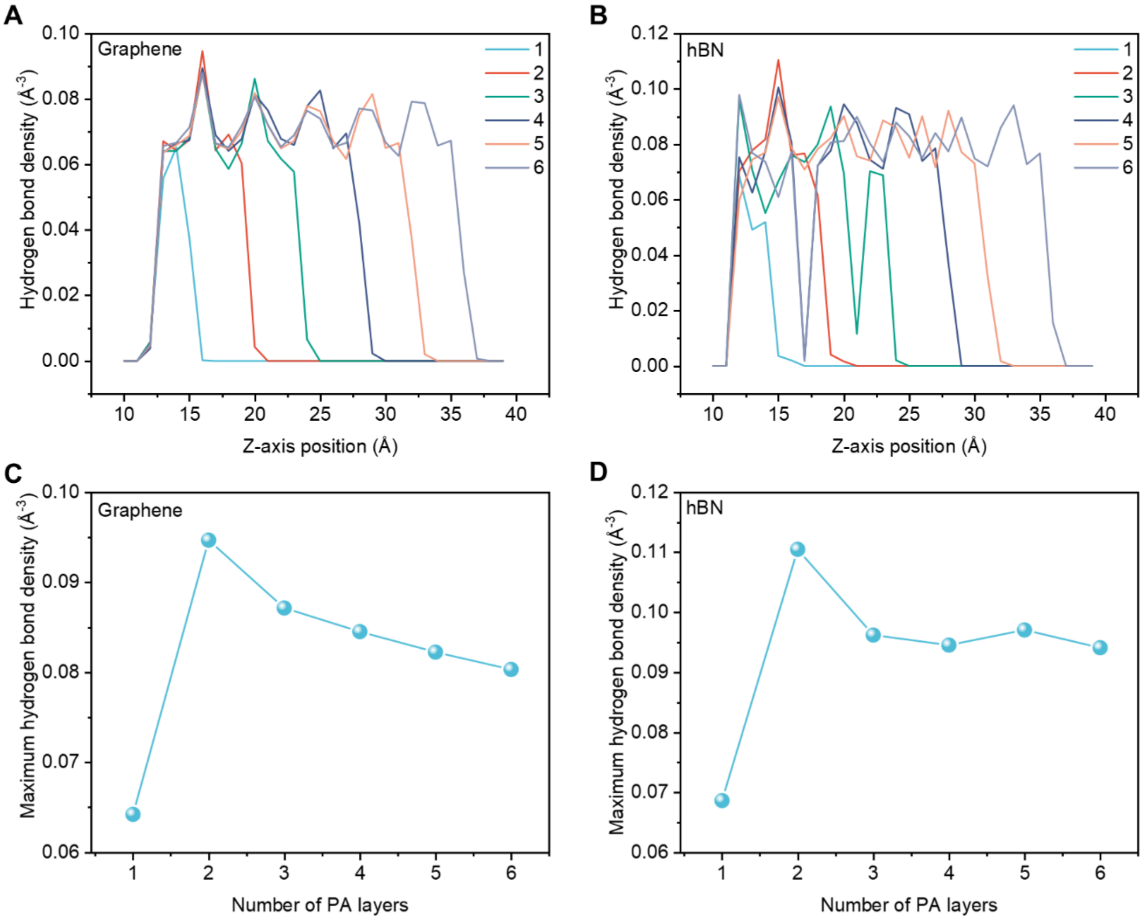

Fig. S34. MD simulation results of PA molecules confined within nanochannels formed by graphene and hBN nanosheets. One-dimensional hydrogen bond density distribution along the Z direction for various numbers of PA layers confined by (**A**) graphene and (**B**) hBN nanosheets. Maximum hydrogen bond density observed with different numbers of PA layers for (**C**) graphene and (**D**) hBN nanosheets. For channels with fewer PA layers, the fixed volume of the vacuum space between the PA layer and the nanosheet occupies a larger proportion relative to the PA volume. As the intersheet distance increases, this vacuum proportion becomes negligible, with the PA molecules occupying the majority of the volume. This leads to a flatter hydrogen bond density profile, indicating that the PA state approaches the bulk state. To further quantify this, the maximum hydrogen bond density values (for cases with more than one PA layer) were plotted for comparison, showing that the two-layer configuration has the highest peak value.

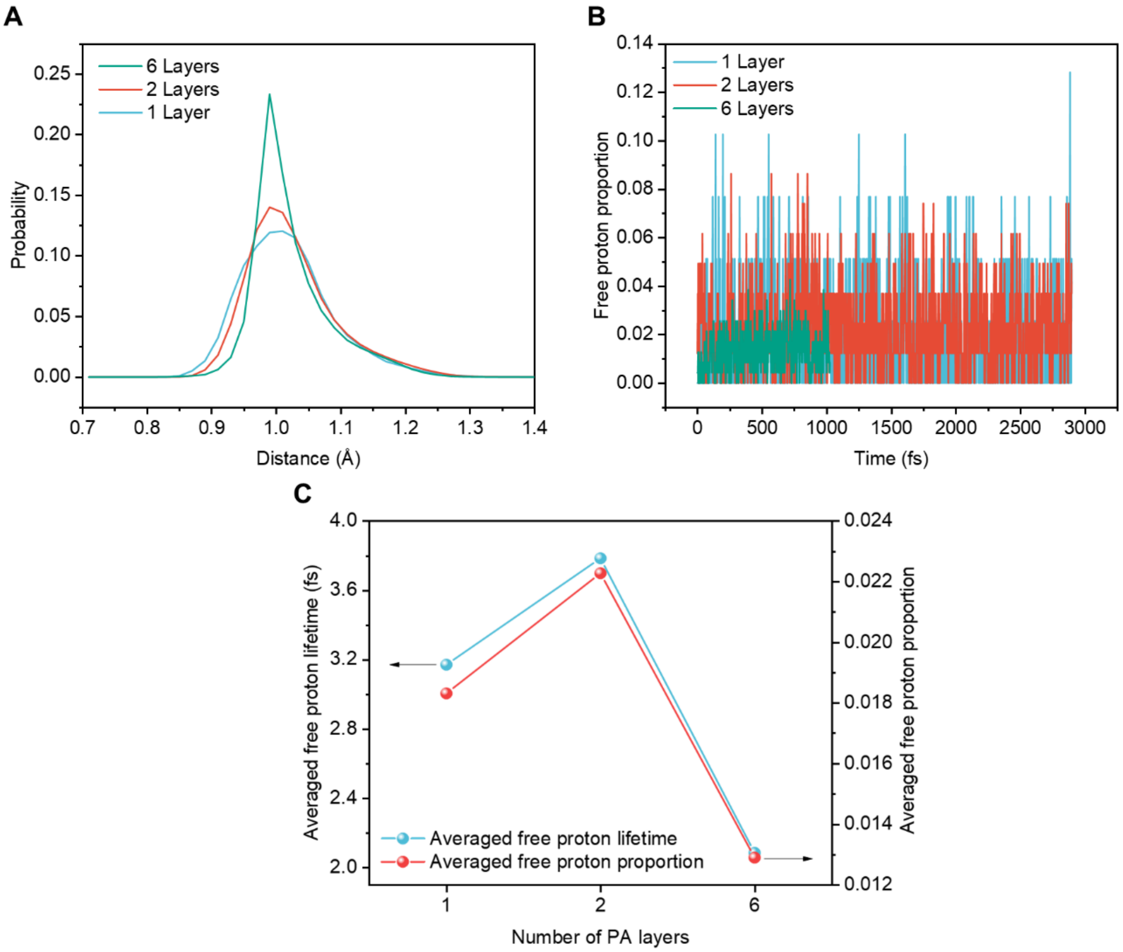

Fig. S35. Free proton lifetime and proportion estimated from MD simulation results for 1, 2, and 6 layers of PA molecules confined within nanochannels formed by graphene nanosheets. (**A**) Distance distribution of protons to their nearest atom. (**B**) Variation in free proton proportion over time. (**C**) Change in average free proton lifetime and proportion with varying numbers of PA layers. A longer free proton lifetime suggests that protons are more likely to hop, while a higher proportion of free protons indicates increased availability for hopping. Thus, the two-layer structure demonstrates the greatest potential for achieving the highest proton conductivity.

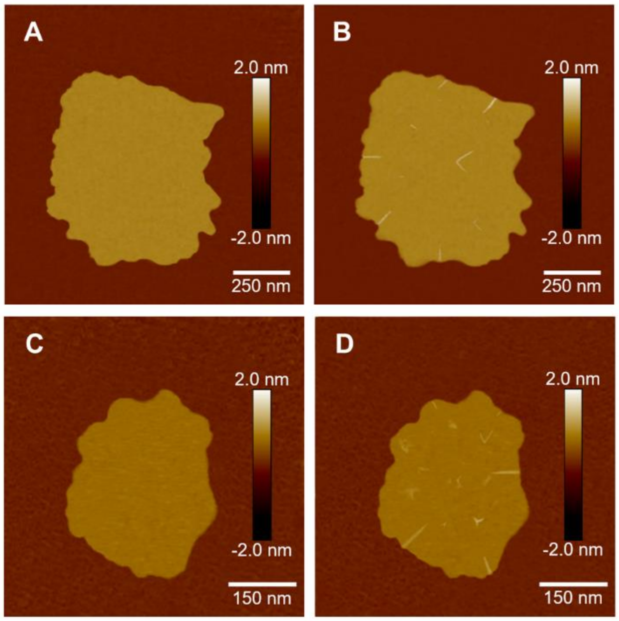

Fig. S 36. AFM images of monolayer graphene nanosheets on Si_3N_4 substrate before (A) and after (B) heating to 250 °C, and AFM images of monolayer BN nanosheets on Si_3N_4 substrate before (C) and after (D) heating to 250 °C.

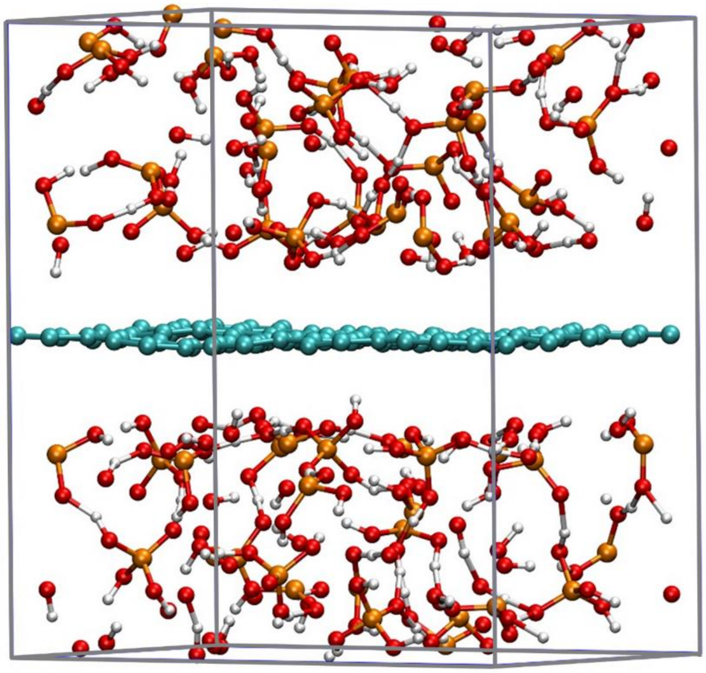

Fig. S37. Demonstration of the Ab initio MD calculation system using graphene as an example. A monolayer graphene is centered in the PA solution with periodic boundary conditions set in all three directions.

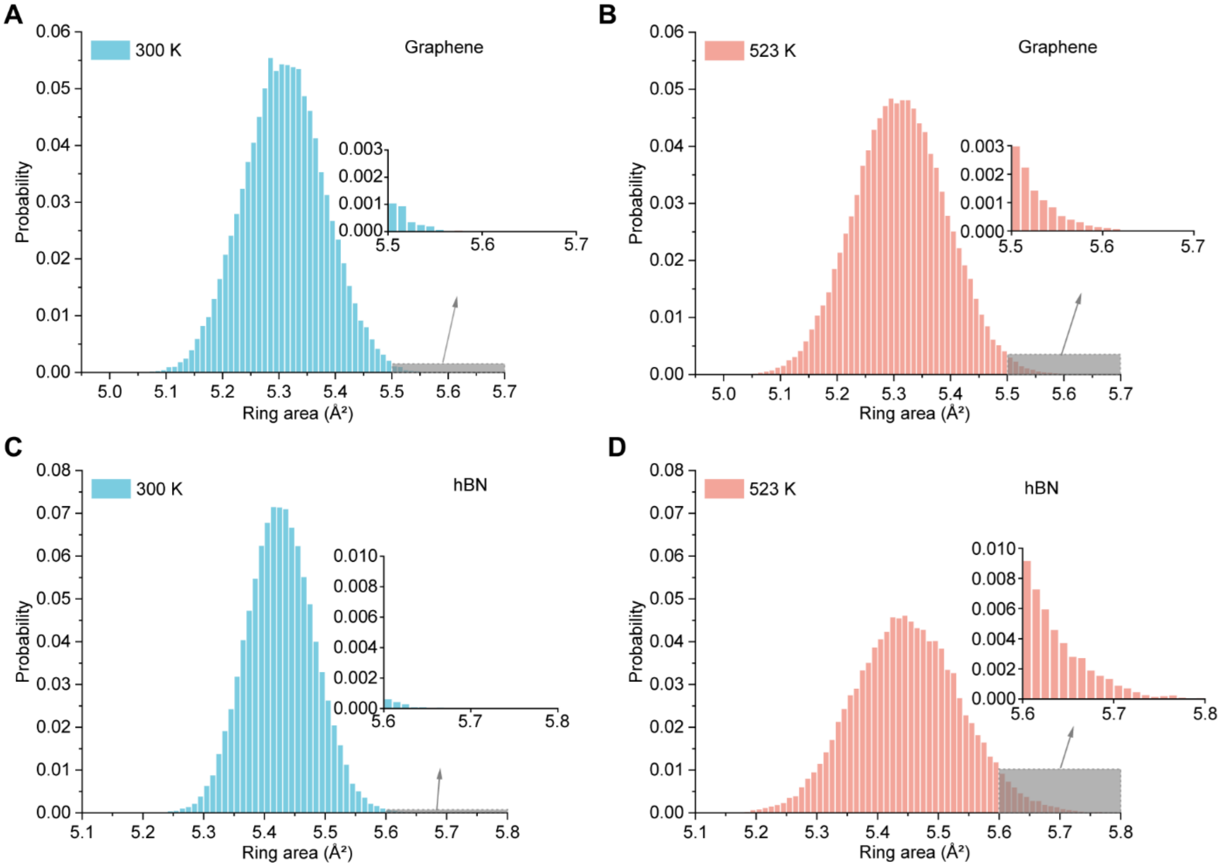

Fig. S38. Ring area distributions of a monolayer graphene nanosheet in PA solution at 300 K (A) and 523 K (B). Ring area distributions of a monolayer hBN nanosheet in PA solution at 300 K (C) and 523 K (D). Monolayer graphene nanosheet exhibits noticeable fluctuations in PA solution at elevated temperatures (Fig. 3I). The transition of nanosheets from flat to curved introduces ring stretching, potentially lowering energy barriers for proton transport through the nanosheet. In fig. S38A and B, the ring areas of graphene nanosheet at both temperatures exhibit Gaussian distributions with nearly identical expectation values, 5.305 and 5.311Å^2 for 300 and 523 K, respectively. However, the distribution width is broader at 523 K, indicating the presence of both larger and smaller ring areas. This trend is similarly observed for hBN in PA solution, as depicted in fig. 38C and D.

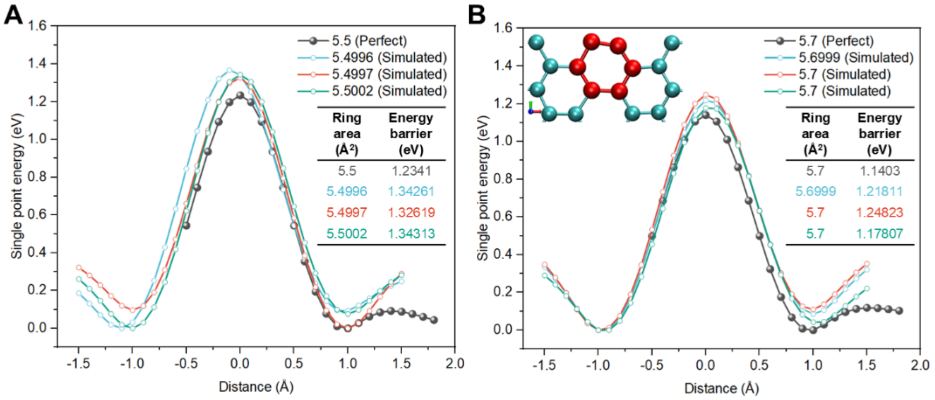

Fig. S39. (A-B) Comparison of energy barrier profiles between the perfect rings and simulated rings with the same ring area. The inset in (B) shows the ring used to calculate the energy barrier profile for the 5.6999 (Simulated) case.

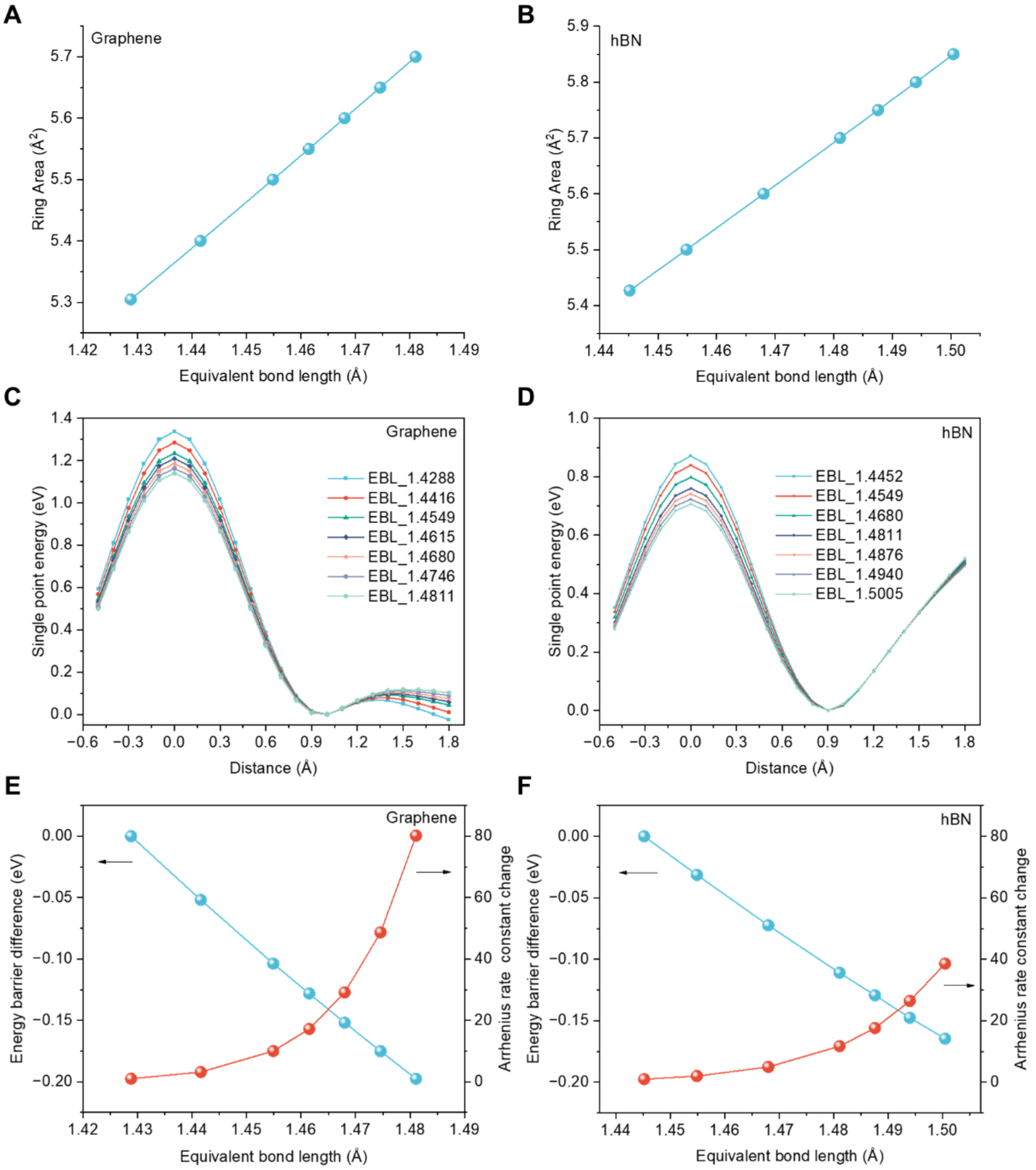

Fig. S40. Correlation between equivalent bond length and ring area for graphene (A) and hBN (B). Energy profiles of a proton located at different distances from monolayer graphene (C) and hBN (D). Variation of energy barrier difference and Arrhenius rate constant change (defined as the ratio of rate constants for a larger ring to the base ring) relating to the equivalent bond length for graphene (E) and hBN (F). The Arrhenius rate constant change was estimated at 523 K. To simplify the analysis, an equivalent bond length was obtained by considering a standard hexagonal ring for a specific ring area, considering the small difference in energy barriers estimated from perfect and simulated rings (fig. S39). For graphene, the equivalent bond lengths for perfect hexagonal rings (all six bond lengths are equal) were calculated across a range of ring areas from 5.305 to 5.70 Å^2. Subsequently, the energy barriers for proton transport through these perfect hexagonal rings were calculated using the DFT method. A similar procedure was implemented for hBN. As the equivalent bond length increases, the energy barrier for through-nanosheet proton transport markedly decreases, correlating with an exponential increase in the Arrhenius rate constant (panels E and F). When a ring on graphene is stretched from a ring area with an equivalent bond length of 1.4288 Å to one with an equivalent bond length of 1.481 Å (panel E), the Arrhenius rate constant experiences an 80-fold increase with only a 3.6 % increase in bond length, substantially boosting the through-nanosheet proton transport.

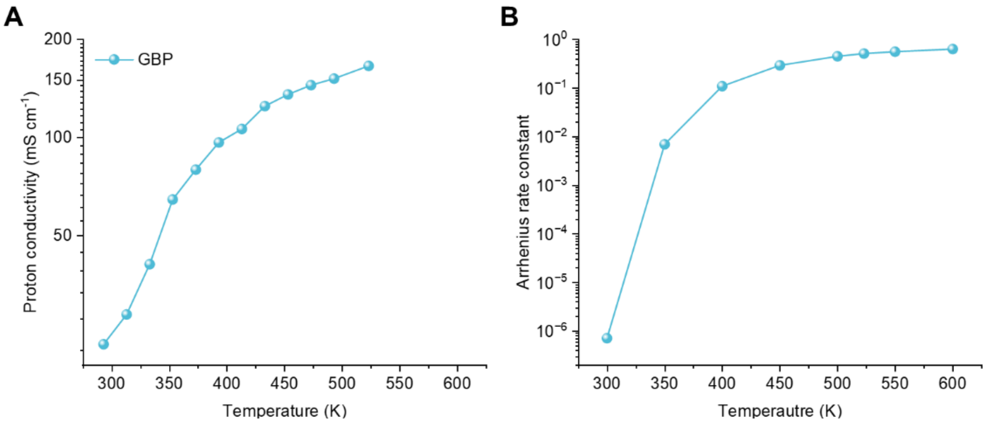

Fig. S41. (A) Proton conductivity of the GBP membrane with 60 wt% PA and 50-µm thickness measured from room temperature to 523 K without external humidification. (B) Temperature dependence of the Arrhenius rate constant corresponding to proton transport through graphene via the quantum tunneling effect.

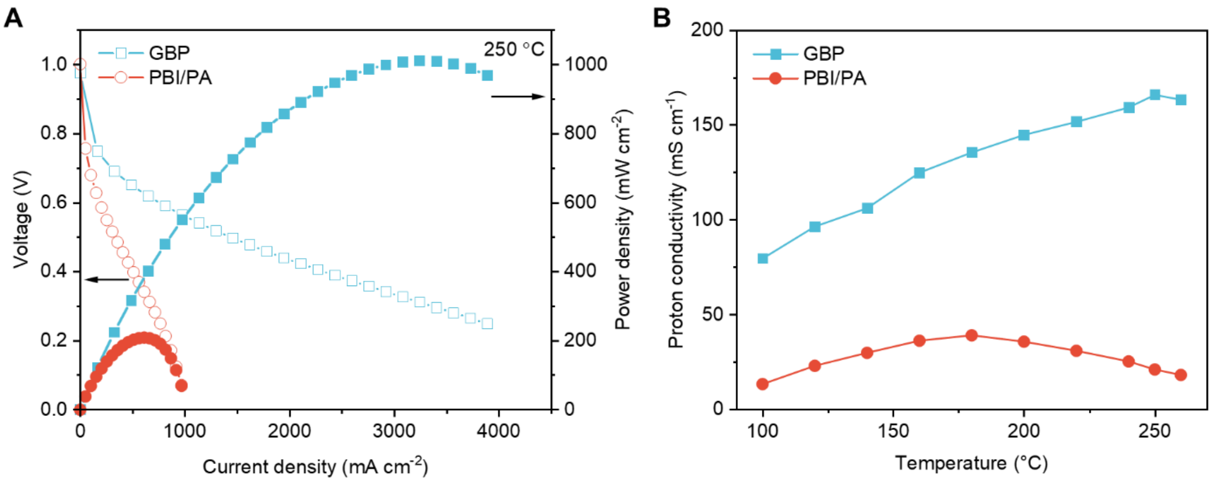

Fig. S42. Comparison of H_2-O_2 fuel cell performance at 250 °C (A) and proton conductivity as a function of temperature (B) between GBP membrane and PBI/PA membrane with 60 wt% PA and 50-µm thickness.

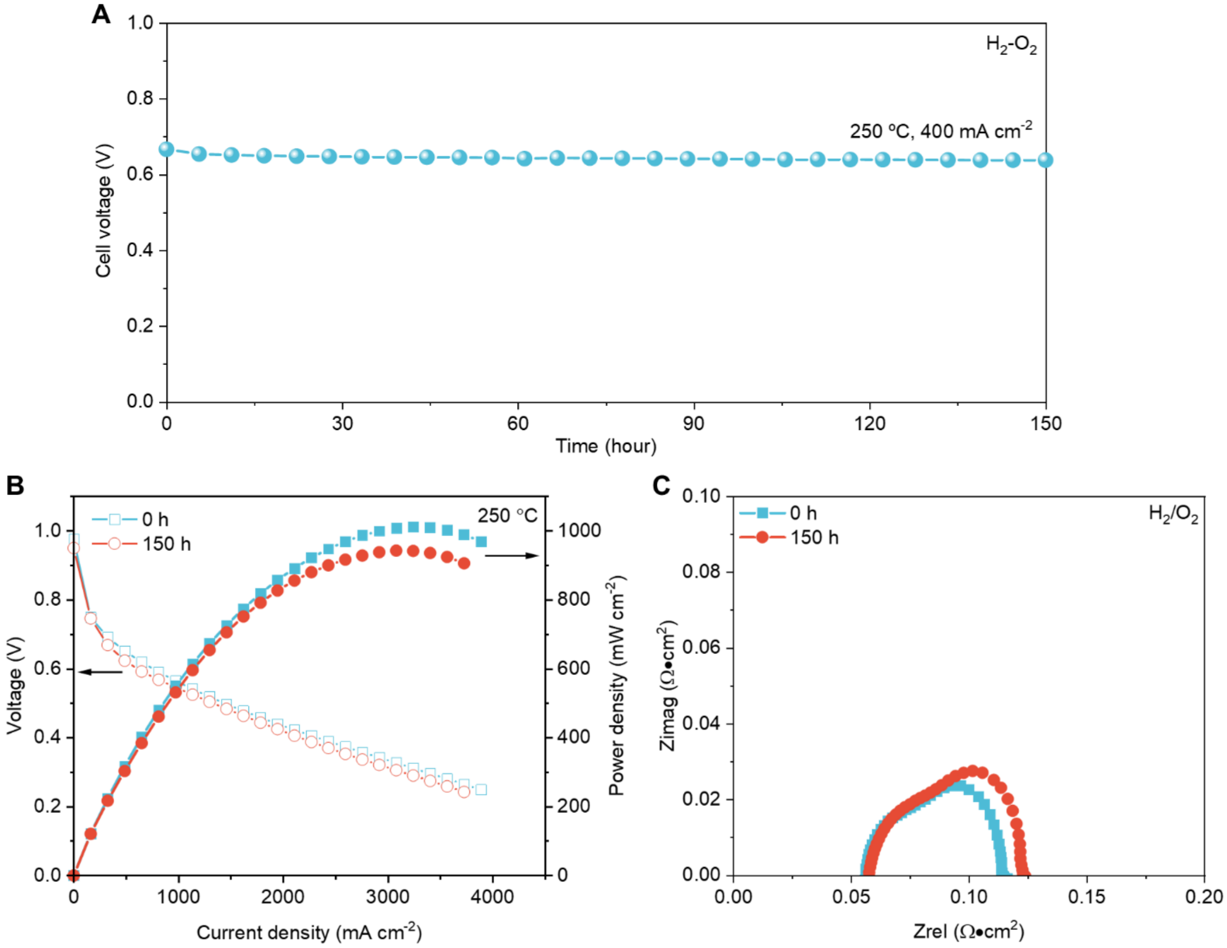

Fig. S43. (A) Operating stability of the H_2-O_2 fuel cell based on 60 wt% PA-doped and 50 µm-thick GBP membrane at a current density of 400 mA cm^{-2} and 250 °C over 150 h. (B) I-V polarization and power density plots, and (C) electrochemical impedance spectra recorded before and after the 150 h operation.

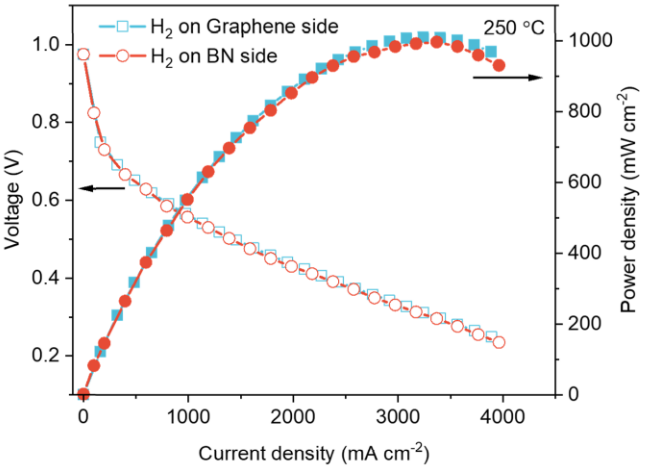

Fig. S44. Comparison of H_2-O_2 fuel cell performance at 250 °C between H_2 flowing on the graphene side and BN side for the GBP membranes with 60 wt% PA and 50-μm thickness.

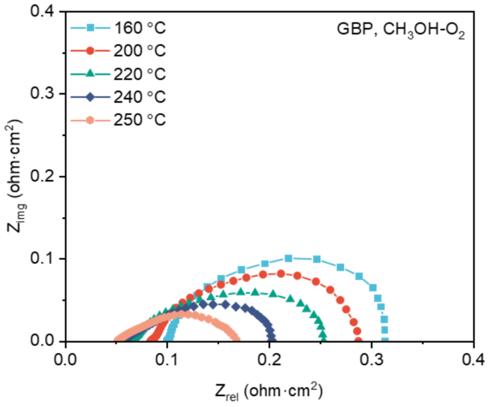

Fig. S45. Electrochemical impedance spectra of the GBP membrane-based DMFC measured at different temperatures and supplied with 16 M methanol and oxygen.

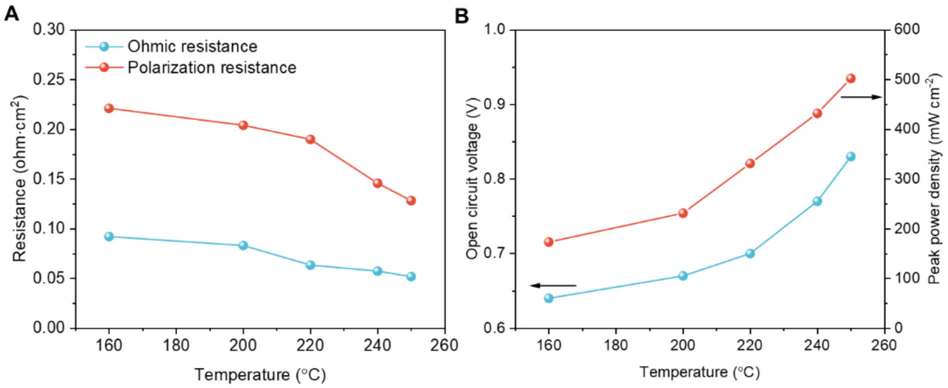

Fig. S46. Temperature-dependent performance of the GBP membrane-based DMFC supplied with 16 M methanol and oxygen. (**A**) Variation of ohmic and polarization resistance with operating temperature. (**B**) Changes in open circuit voltage and peak power density as a function of operating temperature.

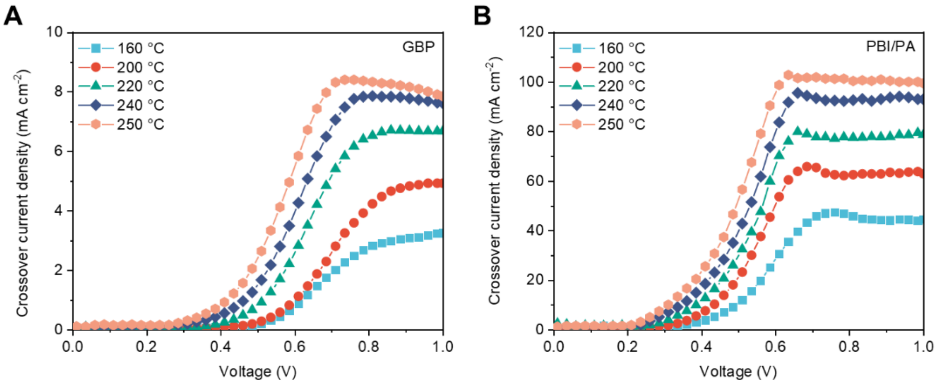

Fig. S47. Variation of methanol crossover current density with applied voltage measured at different temperatures and with the supply of 16 M methanol and dry N_2 for (A) GBP and (B) PBI/PA membrane-based MEAs. Under an applied external potential, methanol is transported from the anode to the cathode, where it undergoes oxidation. The methanol crossover current density is limited by the methanol permeability of the membrane, which was represented by the current density at the plateau of the IV curves around 0.8 V.

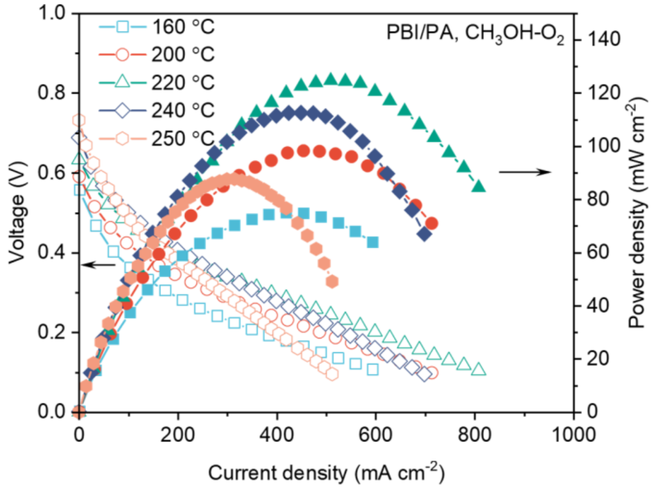

Fig. S48. I-V polarization and power density plots of the PBI/PA membrane-based DMFC measured at different temperatures and supplied with 16 M methanol and oxygen.

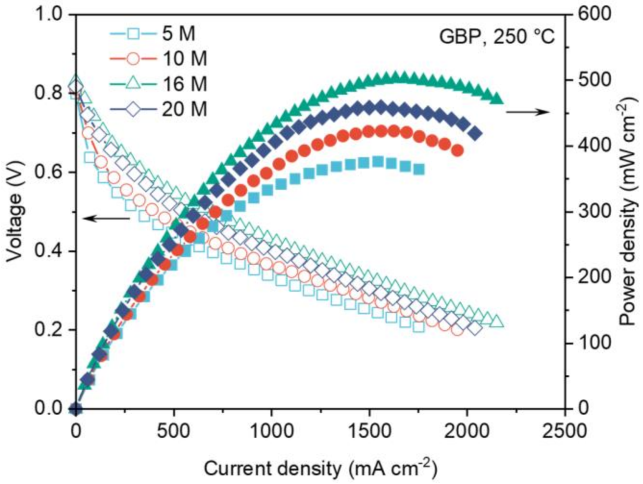

Fig. S49. I-V polarization and power density plots of the GBP membrane-based DMFC measured at 250 °C and supplied with oxygen and methanol at various concentrations.

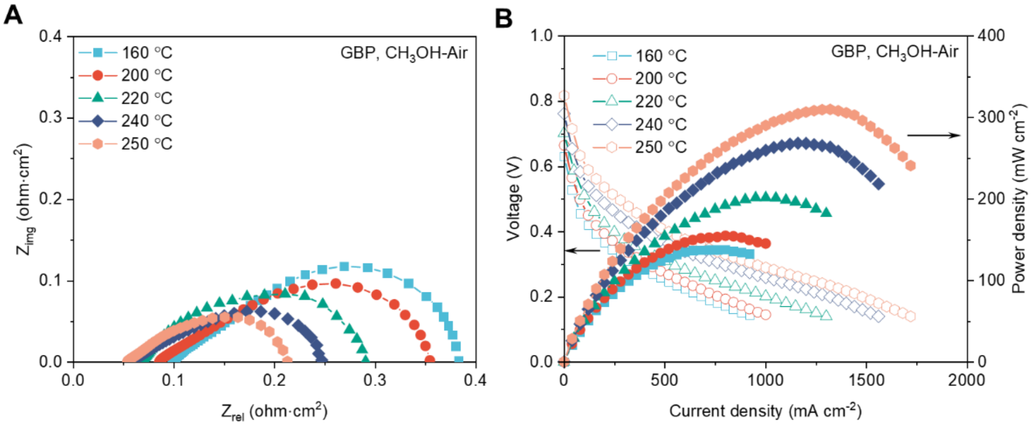

Fig. S50. Electrochemical impedance spectra (A) and I-V polarization and power density plots (B) of the GBP membrane-based DMFC measured at different temperatures and supplied with 16 M methanol and air.

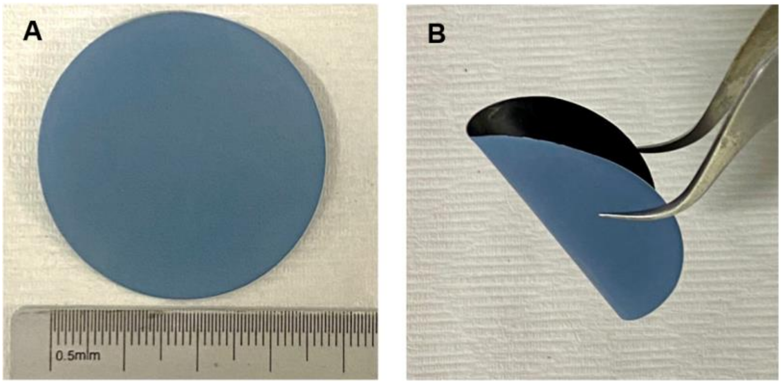

Fig. S51. (A) Photograph of a freestanding GBP membrane with a diameter of ~4 cm. (B) Photograph demonstrating its flexibility when bent with a tweezer.

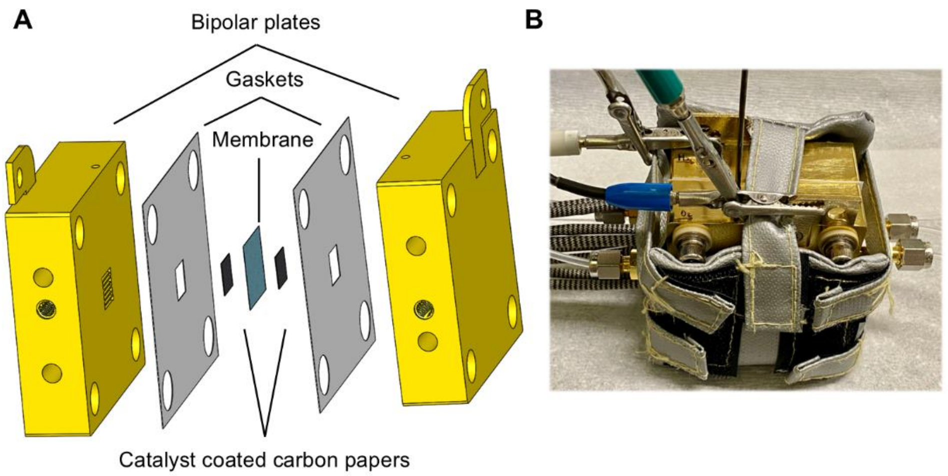

Fig. S52. Images showing (A) membrane-electrode assembly and (B) fuel cell test set-up.

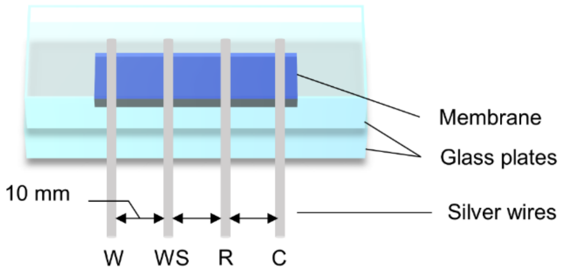

Fig. S53. Illustration showing the set-up for measuring in-plane proton conductivity of the GBP membrane with a four-probe AC impedance method (W-Working electrode, WS-Working sense, R-Reference electrode, and C-Counter electrode).

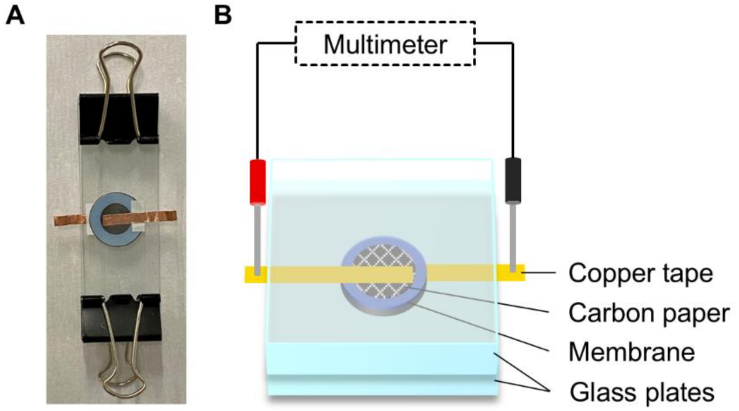

Fig. S54. (A) Photograph and (B) Illustration showing the setup for measuring the electrical conductivity of the GBP membrane.

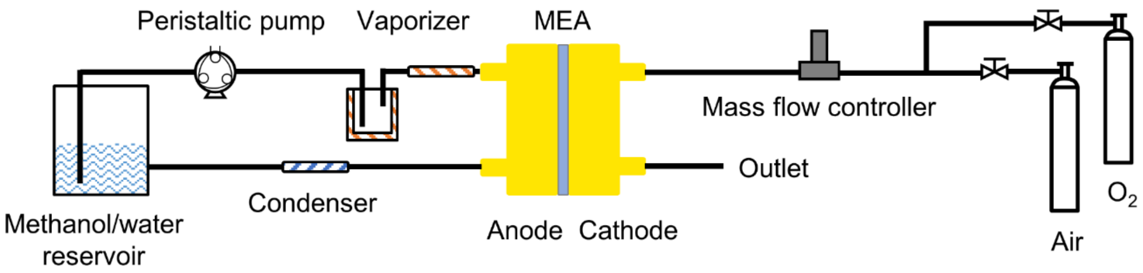

Fig. S55. Schematic diagram showing the set-up for the DMFC test.

Table S1. The weight ratios of elements corresponding to different peaks on the XPS spectra for Graphene/BN (GB) membrane, Graphene/BN/Phosphoric acid (GBP) membrane, and GBP membrane after treatment at 250 °C in the air for 24 h. All XPS results are obtained from the graphene-layer side.

XPS peak	GB membrane	GBP membrane	GBP membrane_250 °C
C1s (sp^2 carbon)	87.54	31.24	30.44
C1s (C-N-H)	8.77	10.66	10.46
N1s ($-NH_2$)	3.70	0.48	0.51
N1s ($-NH^{3+}$)	-	2.27	3.22
P2p (P-O)	-	48.37	48.34
O1s (P-O)	-	6.98	7.03

Table S2. PA loading, *2θ* measured from XRD results, and estimated intersheet spacings of nanosheets of Graphene/BN membranes immersed in PA for different amounts of time.

Immersion time (h)	PA loading (wt%)	*2θ* from XRD (°)	Intersheet spacing (nm)
0	0	26.5	0.34
0.5	25	11.4	0.78
1	43	9.39	0.94
3	60	6.61	1.34
12	67	4.05	2.18
24	78	2.91	3.03

Table S3. Summary of the energy barriers for the GBP membranes with different PA loadings, BN-layer thicknesses, and graphene-layer thicknesses.

PA loading (wt%)	BN-layer thickness (μm)	Graphene-layer thickness (μm)	Energy barrier (kJ mol^{-1})
0	9	41	42.5
25	9	41	12.8
43	9	41	9.9
60	9	41	7.4
67	9	41	8.1
78	9	41	8.5
60	3	41	6.6
60	36	41	8.4
60	80	41	9.7
60	9	15	6.5
60	9	93	9.6

Table S4. Summary of proton conductivity and H_2-O_2 fuel cell performance for the GBP membrane and other membranes recently reported in the literature.

Membranes	Binder	Pt loading (mg cm^{-2})	Test temperature (°C)	Proton conductivity (mS cm^{-1})	Power density (mW cm^{-2})	References
$CsHSO_4$	Naphthalene	18	160	8	11	(*7*)
CsH_2PO_4	Naphthalene	18	235	15	49	(*11*)
CsH_2PO_4	Naphthalene	7.7	240	22	415	(*10*)
HPW/MCM-41	Nafion	0.5	150	45	90	(*8*)
PBI/Polyether/PA	PBI	0.6-0.7	170	120	320	(*54*)
PBI/PDA/PA	PDA-PBI	0.6	160	83	460	(*55*)
PBI/$SnAlP_2O_7$/PA	BASF GDE	1	200	32	440	(*56*)
QAPOH/PA	QASOH	0.6	160	155	738	(*57*)
PIM/PA	PTFE	0.5	160	143	815	(*58*)
PIM/PA	PTFE	0.5	200	133	647	(*58*)
PTFE/BPO_4/PA	PTFE	0.6	250	30	290	(*59*)
PBI/SiO_2/PA	PTFE	1	250	29	289	(*60*)
PES/PVP/HPW/PA	PVP	0.35	160	144	416	(*61*)
Nafion/PA-TPP	QASOH	0.5-0.6	240	79	868	(*13*)
Graphene/BN/PA	PTFE	0.7	160	125	821	This work
Graphene/BN/PA	PTFE	0.7	200	145	919	This work
Graphene/BN/PA	PTFE	0.7	250	166	1011	This work

Table S5. Summary of direct methanol fuel cell performance for the GBP membrane and other advanced membranes.

Membranes	Anode | Cathode catalyst loading	Methanol concentration and flow rate	Test temperature (°C)	Power density (mW cm^{-2})	References
Graphene/BN/PA	1 mg cm^{-2} 50 wt% PtRu/C | 1 mg cm^{-2} 40 wt% Pt/C	16 M, 1 mL min^{-1}	160	173	This work
			200	231	
			220	331	
			240	432	
			250	502	
CsH_2PO_4	13 mg cm^{-2} PtRu | 15 mg cm^{-2} Pt	51 M, 97 mL min^{-1}	243	34	(*11*)
MCM-41/HPW	4 mg cm^{-2} 50 wt% PtRu/C | 1 mg cm^{-2} 50 wt% Pt/C	2 M, 1 mL min^{-1}	110	39	(*8*)
			130	65	
			150	90	
Meso-silica/HPW	4 mg cm^{-2} 50 wt% PtRu/C | 2 mg cm^{-2} 50 wt% Pt/C	2 M, 0.5 mL min^{-1}	80	43	(*62*)
			120	91	
			150	113	
			160	134	
Meso-silica/PA	3 mg cm^{-2} 50 wt% PtRu/C | 2 mg cm^{-2} 50 wt% Pt/C	2 M, 2 mL min^{-1}	170	167	(*63*)
			200	200	
			220	193	
$PBI/SiO_2/PA$	3 mg cm^{-2} 50 wt% PtRu/C | 2 mg cm^{-2} 50 wt% Pt/C	16 M, 0.5 mL min^{-1}	160	108	(*37*)
			200	114	
			220	140	
			230	162	
			240	185	
			250	212	
			260	237	
Nafion/ZrP	2 mg cm^{-2} 45 wt% PtRu/C | 1 mg cm^{-2} 20 wt% Pt/C	5 M, 1 mL min^{-1}	75	96	(*64*)

Nafion/Graphene	2 mg cm^{-2} 40 wt% PtRu/C | 2 mg cm^{-2} 40 wt% Pt/C	2 M, 2 mL min^{-1}	70	118	(*65*)
Nafion/Fullerene	2 mg cm^{-2} 60 wt% PtRu/C | 2 mg cm^{-2} 40 wt% Pt/C	2 M, 2 mL min^{-1}	60	146	(*66*)
SPEEK/Fullerene	2 mg cm^{-2} 60 wt% PtRu/C | 2 mg cm^{-2} 40 wt% Pt/C	2 M, 2 mL min^{-1}	60	103	(*67*)
SPEEK/Zeolite 4A	2 mg cm^{-2} 60 wt% PtRu/C | 2 mg cm^{-2} 40 wt% Pt/C	2 M, 2 mL min^{-1}	70	159	(*68*)
CS/WQAT-4	4 mg cm^{-2} PtRu/C | 2 mg cm^{-2} Pt/C	2 M, 1 mL min^{-1}	70	70	(*69*)
Tokuyama A-006	8 mg cm^{-2} PtRu/C | 8 mg cm^{-2} Pt/C	1 M, 200 mL min^{-1}	90	168	(*70*)
BrPPO/DABCO	5 mg cm^{-2} PtRu/C | 5 mg cm^{-2} Pt/C	4 M, 1 mL min^{-1}	80	132	(*71*)
PVA-b-PVBTAC	4 mg cm^{-2} PtRu/C | 2 mg cm^{-2} Pt/C	1.6 M, 1 mL min^{-1}	60	100	(*72*)

Table S6. A full list of the 23 configurations with their initial intersheet distance, number of PA molecules, and the equilibrium intersheet distance after a 3 ns NVT run. The configurations shown in bold are those selected for subsequent analyses.

**Initial distance d (Å)**	**Number of PA molecules**	**Equilibrium distance d (Å)**
7	9	8.5558
8	12	8.57239
**8.33**	**13**	**8.58115**
8.67	14	8.59861
9	15	8.88945
9.33	16	9.82956
9.67	17	10.5595
10	18	11.07607
11	21	11.8692
12	24	12.46296
**13**	**27**	**12.88249**
14	30	14.2653
15	33	15.62007
16	36	16.25529
**17**	**39**	**16.95297**
18	42	17.31903
19	45	19.23296
20	48	20.14773
**22**	**54**	**21.64817**
24	60	23.98461
**26**	**66**	**25.61921**
28	72	27.72539
**30**	**78**	**29.37907**

Table S7. Parameters for single-point energy calculation in VASP.

SYSTEM	SPE
ISTART	0
ICHARG	2
ENCUT	450
EDIFF	1E-6
EDIFFG	-1E-5
PREC	Normal
ALGO	F
LREAL	Auto
NELM	300
ISMEAR	0
SIGMA	0.2
LWAVE	.FALSE.
LCHARG	.T.
NCORE	8
NELECT	512

REFERENCES

1. K. Jiao, J. Xuan, Q. Du, Z. Bao, B. Xie, B. Wang, Y. Zhao, L. Fan, H. Wang, Z. Hou, S. Huo, N. P. Brandon, Y. Yin, M. D. Guiver, Designing the next generation of proton-exchange membrane fuel cells. *Nature* **595**, 361–369 (2021).

2. R.-T. Liu, Z.-L. Xu, F.-M. Li, F.-Y. Chen, J.-Y. Yu, Y. Yan, Y. Chen, B. Y. Xia, Recent advances in proton exchange membrane water electrolysis. *Chem. Soc. Rev.* **52**, 5652–5683 (2023).

3. A. Ozden, F. P. García de Arquer, J. E. Huang, J. Wicks, J. Sisler, R. K. Miao, C. P. O'Brien, G. Lee, X. Wang, A. H. Ip, E. H. Sargent, D. Sinton, Carbon-efficient carbon dioxide electrolysers. *Nat. Sustain.* **5**, 563–573 (2022).

4. F. Liu, D. Ding, C. Duan, Protonic ceramic electrochemical cells for synthesizing sustainable chemicals and fuels. *Adv. Sci.* **10**, e2206478 (2023).

5. H. Zhang, P. K. Shen, Recent development of polymer electrolyte membranes for fuel cells. *Chem. Rev.* **112**, 2780–2832 (2012).

6. R. Haider, Y. Wen, Z.-F. Ma, D. P. Wilkinson, L. Zhang, X. Yuan, S. Song, J. Zhang, High temperature proton exchange membrane fuel cells: Progress in advanced materials and key technologies. *Chem. Soc. Rev.* **50**, 1138–1187 (2021).

7. S. M. Haile, D. A. Boysen, C. R. I. Chisholm, R. B. Merle, Solid acids as fuel cell electrolytes. *Nature* **410**, 910–913 (2001).

8. S. Lu, D. Wang, S. P. Jiang, Y. Xiang, J. Lu, J. Zeng, HPW/MCM-41 phosphotungstic acid/mesoporous silica composites as novel proton-exchange membranes for elevated-temperature fuel cells. *Adv. Mater.* **22**, 971–976 (2010).

9. Z. Guo, M. Perez-Page, J. Chen, Z. Ji, S. M. Holmes, Recent advances in phosphoric acid–based membranes for high–temperature proton exchange membrane fuel cells. *J. Energy Chem.* **63**, 393–429 (2021).

10. T. Uda, S. M. Haile, Thin-membrane solid-acid fuel cell. *Electrochem. Solid-State Lett.* **8**, A245–A246 (2005).

11. D. A. Boysen, T. Uda, C. R. I. Chisholm, S. M. Haile, High-performance solid acid fuel cells through humidity stabilization. *Science* **303**, 68–70 (2004).

12. Q. Li, J. O. Jensen, R. F. Savinell, N. J. Bjerrum, High temperature proton exchange membranes based on polybenzimidazoles for fuel cells. *Prog. Polym. Sci.* **34**, 449–477 (2009).

13. V. Atanasov, A. S. Lee, E. J. Park, S. Maurya, E. D. Baca, C. Fujimoto, M. Hibbs, I. Matanovic, J. Kerres, Y. S. Kim, Synergistically integrated phosphonated poly(pentafluorostyrene) for fuel cells. *Nat. Mater.* **20**, 370–377 (2021).

14. N. Seselj, D. Aili, S. Celenk, L. N. Cleemann, H. A. Hjuler, J. O. Jensen, K. Azizi, Q. Li, Performance degradation and mitigation of high temperature polybenzimidazole-based polymer electrolyte membrane fuel cells. *Chem. Soc. Rev.* **52**, 4046–4070 (2023).

15. S. Hu, M. Lozada-Hidalgo, F. C. Wang, A. Mishchenko, F. Schedin, R. R. Nair, E. W. Hill, D. W. Boukhvalov, M. I. Katsnelson, R. A. W. Dryfe, I. V. Grigorieva, H. A. Wu, A. K. Geim, Proton transport through one-atom-thick crystals. *Nature* **516**, 227–230 (2014).

16. X. Qian, L. Chen, L. Yin, Z. Liu, S. Pei, F. Li, G. Hou, S. Chen, L. Song, K. H. Thebo, H.-M. Cheng, W. Ren, $CdPS_3$ nanosheets-based membrane with high proton conductivity enabled by Cd vacancies. *Science* **370**, 596–600 (2020).

17. L. Mogg, G.-P. Hao, S. Zhang, C. Bacaksiz, Y.-C. Zou, S. J. Haigh, F. M. Peeters, A. K. Geim, M. Lozada-Hidalgo, Atomically thin micas as proton-conducting membranes. *Nat. Nanotechnol.* **14**, 962–966 (2019).

18. P. R. Kidambi, P. Chaturvedi, N. K. Moehring, Subatomic species transport through atomically thin membranes: Present and future applications. *Science* **374**, eabd7687 (2021).

19. O. J. Wahab, E. Daviddi, B. Xin, P. Z. Sun, E. Griffin, A. W. Colburn, D. Barry, M. Yagmurcukardes, F. M. Peeters, A. K. Geim, M. Lozada-Hidalgo, P. R. Unwin, Proton

transport through nanoscale corrugations in two-dimensional crystals. *Nature* **620**, 782–786 (2023).

20. M. Wang, M. Huang, D. Luo, Y. Li, M. Choe, W. K. Seong, M. Kim, S. Jin, M. Wang, S. Chatterjee, Y. Kwon, Z. Lee, R. S. Ruoff, Single-crystal, large-area, fold-free monolayer graphene. *Nature* **596**, 519–524 (2021).

21. G. He, C. Chang, M. Xu, S. Hu, L. Li, J. Zhao, Z. Li, Z. Li, Y. Yin, M. Gang, H. Wu, X. Yang, M. D. Guiver, Z. Jiang, Tunable nanochannels along graphene oxide/polymer core–shell nanosheets to enhance proton conductivity. *Adv. Funct. Mater.* **25**, 7502–7511 (2015).

22. S. Qin, D. Liu, G. Wang, D. Portehault, C. J. Garvey, Y. Gogotsi, W. Lei, Y. Chen, High and stable ionic conductivity in 2D nanofluidic ion channels between boron nitride layers. *J. Am. Chem. Soc.* **139**, 6314–6320 (2017).

23. M. Komma, A. T. S. Freiberg, D. Abbas, F. Arslan, M. Milosevic, S. Cherevko, S. Thiele, T. Böhm, Applicability of single-layer graphene as a hydrogen-blocking interlayer in low-temperature PEMFCs. *ACS Appl. Mater. Interfaces* **16**, 23220–23232 (2024).

24. J. Chen, J. J. Bailey, L. Britnell, M. Perez-Page, M. Sahoo, Z. Zhang, A. Strudwick, J. Hack, Z. Guo, Z. Ji, P. Martin, D. J. L. Brett, P. R. Shearing, S. M. Holmes, The performance and durability of high-temperature proton exchange membrane fuel cells enhanced by single-layer graphene. *Nano Energy* **93**, 106829 (2022).

25. T. Kim, Y. Sihn, I.-H. Yoon, S. J. Yoon, K. Lee, J. H. Yang, S. So, C. W. Park, Monolayer hexagonal boron nitride nanosheets as proton-conductive gas barriers for polymer electrolyte membrane water electrolysis. *ACS Appl. Nano Mater.* **4**, 9104–9112 (2021).

26. R. N. Karnik, Breakthrough for protons. *Nature* **516**, 173–174 (2014).

27. J. Shen, G. Liu, Y. Han, W. Jin, Artificial channels for confined mass transport at the sub-nanometre scale. *Nat. Rev. Mater.* **6**, 294–312 (2021).

28. A. Falin, Q. Cai, E. J. G. Santos, D. Scullion, D. Qian, R. Zhang, Z. Yang, S. Huang, K. Watanabe, T. Taniguchi, M. R. Barnett, Y. Chen, R. S. Ruoff, L. H. Li, Mechanical properties

of atomically thin boron nitride and the role of interlayer interactions. *Nat. Commun.* **8**, 15815 (2017).

29. Z. Wang, X. Yan, Q. Hou, Y. Liu, X. Zeng, Y. Kang, W. Zhao, X. Li, S. Yuan, R. Qiu, M. H. Uddin, R. Wang, Y. Xia, M. Jian, Y. Kang, L. Gao, S. Liang, J. Z. Liu, H. Wang, X. Zhang, Scalable high yield exfoliation for monolayer nanosheets. *Nat. Commun.* **14**, 236 (2023).

30. Y. Hernandez, V. Nicolosi, M. Lotya, F. M. Blighe, Z. Sun, S. De, I. T. McGovern, B. Holland, M. Byrne, Y. K. Gun'Ko, J. J. Boland, P. Niraj, G. Duesberg, S. Krishnamurthy, R. Goodhue, J. Hutchison, V. Scardaci, A. C. Ferrari, J. N. Coleman, High-yield production of graphene by liquid-phase exfoliation of graphite. *Nat. Nanotechnol.* **3**, 563–568 (2008).

31. C. Korte, "Phosphoric acid, an electrolyte for fuel cells – Temperature and composition dependence of vapor pressure and proton conductivity," in *Fuel Cell Science and Engineering* (John Wiley & Sons Ltd., 2012), pp. 335–359.

32. L. Nie, K. Goh, Y. Wang, J. Lee, Y. Huang, H. E. Karahan, K. Zhou, M. D. Guiver, T.-H. Bae, Realizing small-flake graphene oxide membranes for ultrafast size-dependent organic solvent nanofiltration. *Sci. Adv.* **6**, eaaz9184 (2020).

33. L. Vilčiauskas, M. E. Tuckerman, G. Bester, S. J. Paddison, K.-D. Kreuer, The mechanism of proton conduction in phosphoric acid. *Nat. Chem.* **4**, 461–466 (2012).

34. I. Poltavsky, L. Zheng, M. Mortazavi, A. Tkatchenko, Quantum tunneling of thermal protons through pristine graphene. *J. Chem. Phys.* **148**, 204707 (2018).

35. M. Ahmed, I. Dincer, A review on methanol crossover in direct methanol fuel cells: Challenges and achievements. *Int. J. Energy Res.* **35**, 1213–1228 (2011).

36. J. R. Varcoe, P. Atanassov, D. R. Dekel, A. M. Herring, M. A. Hickner, P. A. Kohl, A. R. Kucernak, W. E. Mustain, K. Nijmeijer, K. Scott, T. Xu, L. Zhuang, Anion-exchange membranes in electrochemical energy systems. *Energ. Environ. Sci.* **7**, 3135–3191 (2014).

37. Y. Cheng, J. Zhang, S. Lu, S. P. Jiang, Significantly enhanced performance of direct methanol fuel cells at elevated temperatures. *J. Power Sources* **450**, 227620 (2020).

38. H. Liu, C. Song, L. Zhang, J. Zhang, H. Wang, D. P. Wilkinson, A review of anode catalysis in the direct methanol fuel cell. *J. Power Sources* **155**, 95–110 (2006).

39. K. He, Y. Hu, Z.-X. Low, R. Wang, F. Wang, H. Ma, X. Chen, D. R. MacFarlane, H. Wang, Metal oxyhydroxide nanosheet-assisted fabrication of ultrathin carbon molecular sieve membrane for hydrogen separation. *J. Mater. Chem. A* **10**, 18095–18102 (2022).

40. L. Martínez, R. Andrade, E. G. Birgin, J. M. Martínez, PACKMOL: A package for building initial configurations for molecular dynamics simulations. *J. Comput. Chem.* **30**, 2157–2164 (2009).

41. Y. Wang, A. Kiziltas, P. Blanchard, T. R. Walsh, Calculation of 1D and 2D densities in VMD: A flexible and easy-to-use code. *Comput. Phys. Commun.* **266**, 108032 (2021).

42. S. Plimpton, Fast parallel algorithms for short-range molecular dynamics. *J. Comput. Phys.* **117**, 1–19 (1995).

43. K. Vanommeslaeghe, E. Hatcher, C. Acharya, S. Kundu, S. Zhong, J. Shim, E. Darian, O. Guvench, P. Lopes, I. Vorobyov, A. D. Mackerell, CHARMM general force field: A force field for drug-like molecules compatible with the CHARMM all-atom additive biological force fields. *J. Comput. Chem.* **31**, 671–690 (2010).

44. W. L. Jorgensen, Pressure dependence of the structure and properties of liquid n-butane. *J. Am. Chem. Soc.* **103**, 4721–4726 (1981).

45. Y. Wang, Y. Wang, HBCalculator: A tool for hydrogen bond distribution calculations in molecular dynamics simulations. *J. Chem. Inf. Model.* **64**, 1772–1777 (2024).

46. A. K. Soper, M. A. Ricci, Structures of high-density and low-density water. *Phys. Rev. Lett.* **84**, 2881–2884 (2000).

47. A. Nilsson, L. G. M. Pettersson, The structural origin of anomalous properties of liquid water. *Nat. Commun.* **6**, 8998 (2015).

48. P. A. Sigala, E. A. Ruben, C. W. Liu, P. M. B. Piccoli, E. G. Hohenstein, T. J. Martínez, A. J. Schultz, D. Herschlag, Determination of hydrogen bond structure in water versus aprotic environments to test the relationship between length and stability. *J. Am. Chem. Soc.* **137**, 5730–5740 (2015).

49. G. Kresse, J. Furthmüller, Efficiency of ab-initio total energy calculations for metals and semiconductors using a plane-wave basis set. *Comput. Mater. Sci.* **6**, 15–50 (1996).

50. Y. Wang, K. He, D. Dong, J. Gu, J. Z. Liu, Y. Wang, HexagonRingCalculator: A handy code for hexagonal ring characterization in atomistic simulations. *J. Chem. Inf. Model.* **64**, 7827–7832 (2024).

51. K. Li, Z. Bo, J. Yan, K. Cen, Solid-state NMR study of ion adsorption and charge storage in graphene film supercapacitor electrodes. *Sci. Rep.* **6**, 39689 (2016).

52. A. C. Forse, J. M. Griffin, V. Presser, Y. Gogotsi, C. P. Grey, Ring current effects: Factors affecting the NMR chemical shift of molecules adsorbed on porous carbons. *J. Phys. Chem. C* **118**, 7508–7514 (2014).

53. S. Chandra, T. Kundu, S. Kandambeth, R. BabaRao, Y. Marathe, S. M. Kunjir, R. Banerjee, Phosphoric acid loaded azo (−N═N−) based covalent organic framework for proton conduction. *J. Am. Chem. Soc.* **136**, 6570–6573 (2014).

54. Q. Li, J. O. Jensen, C. Pan, V. Bandur, M. S. Nilsson, F. Schönberger, A. Chromik, M. Hein, T. Häring, J. Kerres, N. J. Bjerrum, Partially fluorinated aarylene polyethers and their ternary blends with PBI and H_3PO_4. Part II. Characterisation and fuel cell tests of the ternary membranes. *Fuel Cells* **8**, 188–199 (2008).

55. J. Fang, X. Lin, D. Cai, N. He, J. Zhao, Preparation and characterization of novel pyridine-containing polybenzimidazole membrane for high temperature proton exchange membrane fuel cells. *J. Membr. Sci.* **502**, 29–36 (2016).

56. Y. C. Jin, M. Nishida, W. Kanematsu, T. Hibino, An H_3PO_4-doped polybenzimidazole/ $Sn_{0.95}Al_{0.05}P_2O_7$ composite membrane for high-temperature proton exchange membrane fuel cells. *J. Power Sources* **196**, 6042–6047 (2011).

57. K.-S. Lee, J. S. Spendelow, Y.-K. Choe, C. Fujimoto, Y. S. Kim, An operationally flexible fuel cell based on quaternary ammonium-biphosphate ion pairs. *Nat. Energy* **1**, 1–7 (2016).

58. H. Tang, K. Geng, L. Wu, J. Liu, Z. Chen, W. You, F. Yan, M. D. Guiver, N. Li, Fuel cells with an operational range of −20 °C to 200 °C enabled by phosphoric acid-doped intrinsically ultramicroporous membranes. *Nat. Energy* **7**, 153–162 (2022).

59. R. Lan, X. Xu, S. Tao, J. T. S. Irvine, A fuel cell operating between room temperature and 250°C based on a new phosphoric acid based composite electrolyte. *J. Power Sources* **195**, 6983–6987 (2010).

60. Y. Cheng, J. Zhang, S. Lu, H. Kuang, J. Bradley, R. De Marco, D. Aili, Q. Li, C. Q. Cui, S. P. Jiang, High CO tolerance of new SiO_2 doped phosphoric acid/polybenzimidazole polymer electrolyte membrane fuel cells at high temperatures of 200–250 °C. *Int. J. Hydrogen Energy* **43**, 22487–22499 (2018).

61. J. Zhang, S. Chen, H. Bai, S. Lu, Y. Xiang, S. P. Jiang, Effects of phosphotungstic acid on performance of phosphoric acid doped polyethersulfone-polyvinylpyrrolidone membranes for high temperature fuel cells. *Int. J. Hydrogen Energy* **46**, 11104–11114 (2021).

62. J. Zeng, S. P. Jiang, Characterization of high-temperature proton-exchange membranes based on phosphotungstic acid functionalized mesoporous silica nanocomposites for fuel cells. *J. Phys. Chem. C* **115**, 11854–11863 (2011).

63. J. Zeng, B. He, K. Lamb, R. D. Marco, P. K. Shen, S. P. Jiang, Phosphoric acid functionalized pre-sintered meso-silica for high temperature proton exchange membrane fuel cells. *Chem. Commun.* **49**, 4655–4657 (2013).

64. H. Hou, G. Sun, Z. Wu, W. Jin, Q. Xin, Zirconium phosphate/Nafion115 composite membrane for high-concentration DMFC. *Int. J. Hydrogen Energy* **33**, 3402–3409 (2008).

65. V. Parthiban, S. Akula, S. G. Peera, N. Islam, A. K. Sahu, Proton conducting nafion-sulfonated graphene hybrid membranes for direct methanol fuel cells with reduced methanol crossover. *Energy Fuel* **30**, 725–734 (2016).

66. G. Rambabu, N. Nagaraju, S. D. Bhat, Functionalized fullerene embedded in Nafion matrix: A modified composite membrane electrolyte for direct methanol fuel cells. *Chem. Eng. J.* **306**, 43–52 (2016).

67. G. Rambabu, S. D. Bhat, Sulfonated fullerene in SPEEK matrix and its impact on the membrane electrolyte properties in direct methanol fuel cells. *Electrochim. Acta* **176**, 657–669 (2015).

68. S. Meenakshi, S. D. Bhat, A. K. Sahu, P. Sridhar, S. Pitchumani, Modified sulfonated poly(ether ether ketone) based mixed matrix membranes for direct methanol fuel cells. *Fuel Cells* **13**, 851–861 (2013).

69. W.-C. Tsen, Composite proton exchange membranes based on chitosan and phosphotungstic acid immobilized one-dimensional attapulgite for direct methanol fuel cells. *Nanomaterials* **10**, 1641 (2020).

70. G. K. S. Prakash, F. C. Krause, F. A. Viva, S. R. Narayanan, G. A. Olah, Study of operating conditions and cell design on the performance of alkaline anion exchange membrane based direct methanol fuel cells. *J. Power Sources* **196**, 7967–7972 (2011).

71. A. Katzfuß, V. Gogel, L. Jörissen, J. Kerres, The application of covalently cross-linked BrPPO as AEM in alkaline DMFC. *J. Membr. Sci.* **425-426**, 131–140 (2013).

72. M. Higa, S. Mehdizadeh, S. Feng, N. Endo, Y. Kakihana, Cell performance of direct methanol alkaline fuel cell (DMAFC) using anion exchange membranes prepared from PVA-Based block copolymer. *J. Membr. Sci.* **597**, 117618 (2020).
